# Supplementary material for: Household alternating current electricity plug-and-play quantum-dot light-emitting diodes
Source: Nat Commun. 2024 Apr 25;15:3512. doi: 10.1038/s41467-024-47891-4 (PMC11045821; doi:10.1038/s41467-024-47891-4)
Supplement: Supplementary file 1 — Supplementary Information [file 41467_2024_47891_MOESM1_ESM.pdf]

Supplementary Information

Household alternating current electricity plug-and-play quantum-dot light-emitting diodes

Jiming Wang[1, 2], Cuixia Yuan[1], Shuming Chen[1,*]

[1] Department of Electrical and Electronic Engineering, Southern University of Science and Technology, Shenzhen, 518055, P. R. China

[2] Harbin Institute of Technology, Harbin, 150001, P. R. China

* Corresponding author: Shuming Chen (chen.sm@sustech.edu.cn)

Supplementary Movie

Supplementary Movie 1 A tandem QLED with B-QLED and T-QLED connected in in parallel. Driven by an AC source, both B-QLED and T-QLED are alternately turned on.

Supplementary Movie 2 A basis PnP-QLED with two tandem QLEDs connected in series. Driven by an AC source, the B-QLED (or T-QLED) of the first tandem device and the T-QLED (or B-QLED) of the second tandem device are simultaneously turned on.

Supplementary Movie 3 A $(PnP\text{-}QLED)_{30}$ with 30 tandem QLEDs connected in series. By increasing the AC voltage level, the brightness of the device is gradually increased. Note that the observable flicker is due to the mismatch of the QLED lighting frequency and the frame captured frequency of the camera. The emission is quite stable observed by eyes.

Supplementary Movie 4 The developed red (n=30), yellow (n=28), and white (n=26) $(PnP\text{-}QLED)_n$ can be directly plugged into a household 220 V/50 Hz power supply without needing any accessories. Note that the observable flicker is due to the mismatch of the QLED lighting frequency and the frame captured frequency of the camera. The emission is quite stable observed by eyes.

Supplementary Figures and Tables

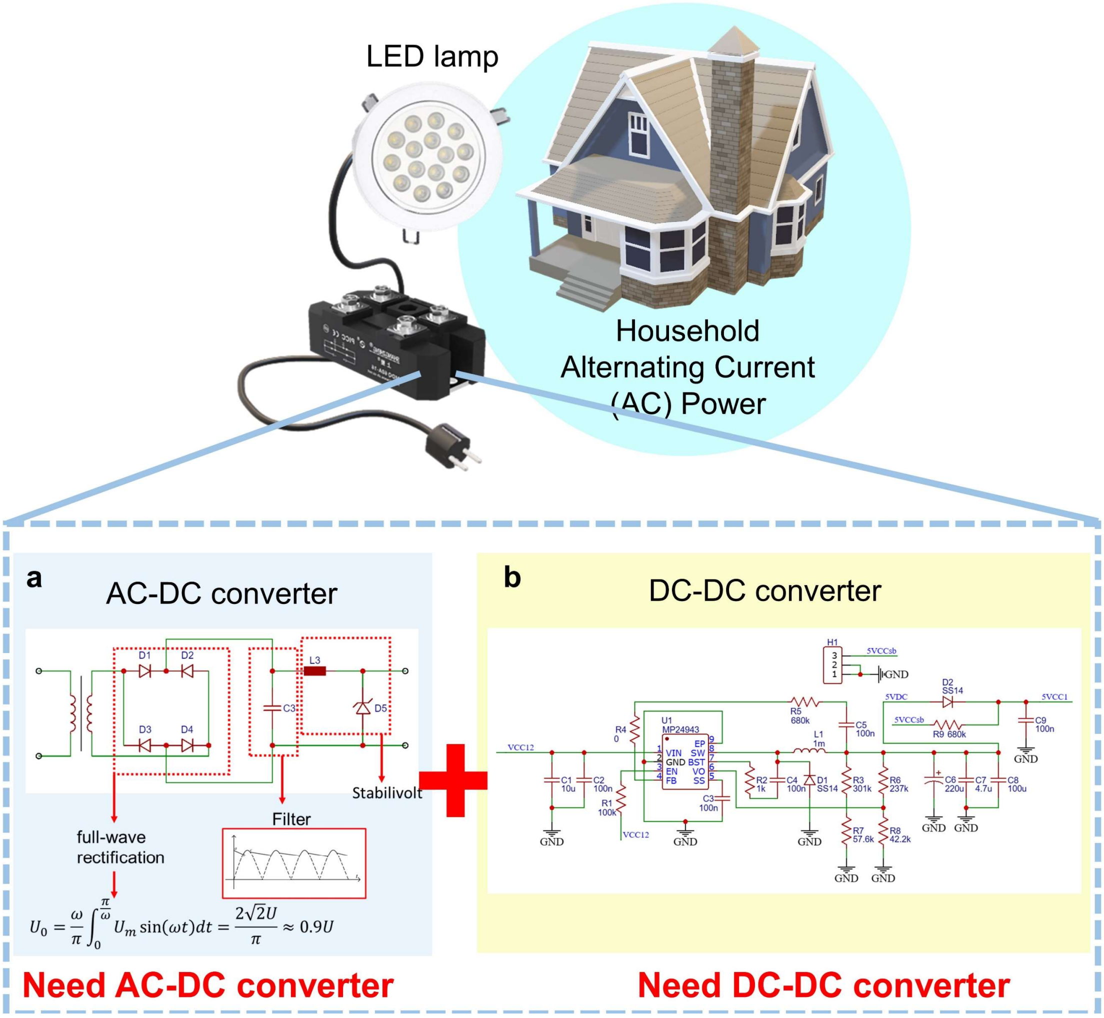

Supplementary Fig. 1 Composition of LED switching power supply. a, AC-DC converter: A device that converts household alternating current (AC) electricity into direct current (DC).[1,2] **b**, DC-DC converter: A device that transforms direct current (DC) into another form of direct current (DC), such as using this device to convert high voltage and low current to low voltage and high current.[3,4]

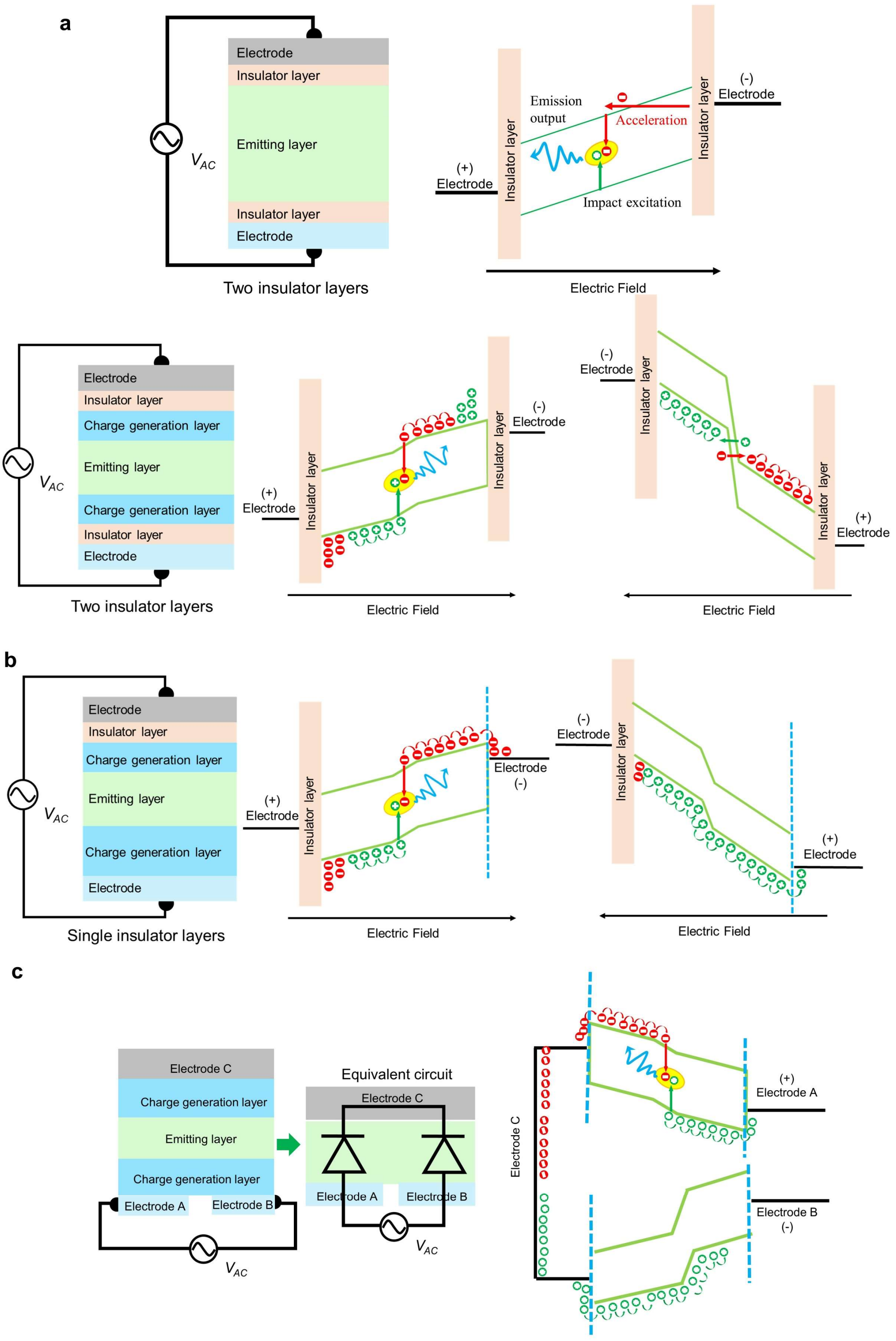

Supplementary Fig. 2 Structure and principle of AC-EL devices. a-c, Device structure and working principle of AC-EL devices with two insulating layers (**a**), single insulating layer (**b**), and coplanar electrodes (**c**), respectively.

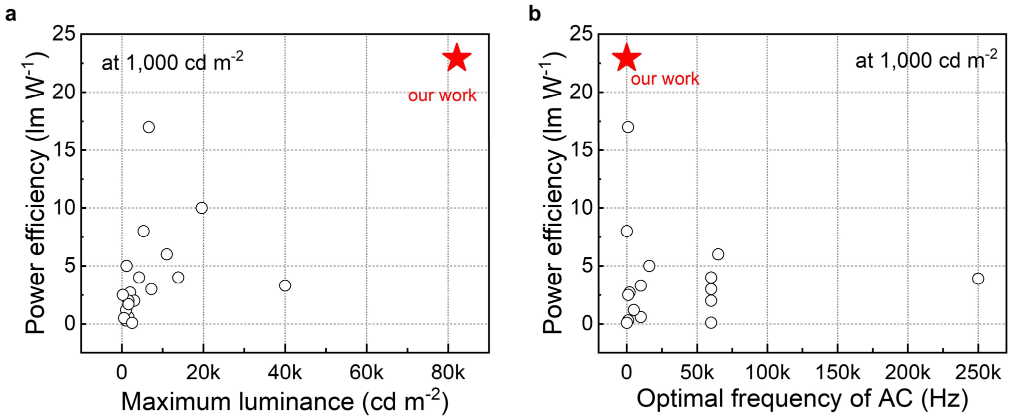

Supplementary Fig. 3 Power efficiency comparison of reported AC driven devices at 1,000 cd m^{-2}.[5-25] See also Supplementary Table 1. **a**, Power efficiency at 1,000 cd m^{-2} as a function of maximum luminance of AC driven devices. **b**, Power efficiency at 1,000 cd m^{-2} as a function of optimal frequency of AC driven devices.

Supplementary Table 1 Performances of AC-driven EL devices

Type	Device architecture	EL (nm)	Power efficiency at 1,000 cd m^{-2} (lm W^{-1})	Max Luminance (cd m^{-2})	Max V_{RMS} (V)	Optimal Frequency (Hz)	Response of EL in AC voltage cycle	Compatible with the household AC power lines	Ref
Two insulating layers	ITO/Al_2O_3/ZnS/ZnSe/ZnS:Mn/ ZnS/ Al_2O_3/ITO	Orange	N/A	N/A	170	30,000	Full cycle	NO	5
	ITO/SiO_2/MeO-TPD:F4TCNQ/ Ir(MDQ)$_2$(acac)/Bphen:Cs/SiO_2/Al	610	< 0.3	~1,000	~70	~1,000	Half cycle	NO	6
	ITO/HfO_2 / MeO-TPD:F4TCNQ/NPB/ Ir(MDQ)$_2$(acac) / Bphen/ Bphen:Cs/HfO_2/Al	610	0.6	1,581	23	10,000	Half cycle	NO	7
	ITO/LiF/MoO_3:NPB/NPB/Bphen/ Bphen:Cs_2CO_3/LiF/Al	516	N/A	87	15	10,000	Half cycle	NO	8

	ITO/HfO_2 / MeO-TPD:F6TCNQ/Spiro-TAD/ α-NPD Ir(MDQ)$_2$(acac) / Bphen/ Bphen:Cs/HfO_2/Al	610	~1.2	1,000	40	5,000	Half cycle	NO	[9]
	ITO/HfO_2 / MeO-TPD:F6TCNQ/Spiro-TAD/TCTA:Ir(ppy)$_3$/ Bphen/ Bphen:Cs/HfO_2/Al	518	2.7 (under 500 cd m^{-2})	2,000	26.5	2,000	Half cycle	NO	[10]
Single insulating layer	Ag/$BaTiO_3$/ZnS:Cu/Graphene	Cyan	5.0	1,140	480	16,000	Full cycle	NO	[11]
	ITO/SiO_2/F8BT/Au	550	N/A	650	25	300,000	Half cycle	NO	[12]
	ITO/SiO_2/PEDOT:PSS/MWNTs nanocomposite/LiF/Al	550	3.3	40,000	45	10,000	Half cycle	NO	[13]
	ITO/SiO_2/F8BT or SPR-001 or SPR-02T/Ag	540	0.6 cd A^{-1}	954	60	50,000	Half cycle	NO	[14]

		White (peak 538)	N/A	5,984	80	300,000			
	ITO/P(VDF-TrFE-CFE)/MoO_3/TFB/QD/ZnMgO/Al	534	3.9 cd A^{-1}	65,760	60	250,000	Half cycle	NO	15
	ITO/Ferroelectric dielectric polymers /Poly-TPD:F4TCN/PVK:OXD-7:$Ir(ppy)_3$/TmPyPB /LiF/Al	~510	6.0	10,995	~120	65,000	Half cycle	NO	16
	ITO/PF-BT-QL:$Ir(MDQ)_2(acac)$/ P(VDF-TrFE) /Al	White	0.11 (under 87 cd m^{-2})	87	~160	60,000	Half cycle	NO	17
	ITO/P (VDF-TrFE)75-25 /Poly-TPD:F4TCNQ/PVK:OXD-7:Phosphors/TmPyPB /LiF/Al	472	~2.0	3,000	140	60,000	Half cycle	NO	18
		516	~4.0	13,800	120				

		590	~2.0	1,600	140				
	ITO/SWNTs:P (VDF-TrFE-CFE) nanocomposites /P3HT:F4TCNQ/PVK:OXD-7 (80:20):FIrpic:Ir(ppy)$_3$:Ir(MDQ)$_2$(acac) /TmPyPB:Li_2CO_3/LiF/Al	White	~3.0	7,210	100	60,000	Half cycle	NO	[19]
Coplanar electrodes	Mg:Ag/MoO_3/TAPC/PO-01:FirPic/BPhen/Liq/Ag side-by-side Mg:Ag/MoO_3/TAPC/ PO-01:FirPic /BPhen/Liq/Ag	Blue to white to yellow	~35 (yellow) and ~10 (blue)	11,153 (blue) and 19,563 (yellow)	10 (blue) and 11 (yellow)	50	Full cycle	NO	[20]
	ITO/P(VDF-TrFE)/HATCN/TAPC/26DCzPPy:FirPic/Bphen/LiF/Al side-by-side ITO/P(VDFTrFE)/HATCN/TAPC/26DCzPPy:FirPic /Bphen/LiF/Al	Blue to white to yellow	< 0.5 (blue) <1.7 (yellow)	500 (blue) 1,600 (yellow)	110	1,000	Full cycle	NO	[21]

	ITO/ZnO/PEI/PDY132/MWNT/ PEDOT:PSS /SiO_2/Al side-by-side ITO/ZnO/PEI/PDY132/MWNT/ PEDOT:PSS /SiO_2/Al	Yellow	N/A	2,000	60	400,000	Full cycle	NO	[22]
	ITO/MoO_3/TCTA/26DCzPPy: FirPic / Bphen:Ag/LiF / Mg:Ag/LiF/Bphen:Ag/CBP:PO-01/TCTA/MoO_3/ITO	Blue and yellow	~4.0 (blue) and ~8.0 (yellow)	4,200 (blue) and 5,300 (yellow)	20	50	Full cycle	NO	[23]
	ITO/PEDOT:PSS/ZnO/QD/ZnO/PEDOT:PSS /Al	635 and 524	< 0.1	2,492	20	50	Full cycle	NO	[24]
	ITO/$BaTiO_3$/ZnS:Cu/Polar bridge side-by-side ITO/$BaTiO_3$/ZnS:Cu/Polar bridge side-by-side ITO/$BaTiO_3$/ZnS:Cu/Polar bridge	White	~2.5	215	120	1,000	Full cycle	NO	[25]
	ITO/PEDOT:PSS/P(VDF-TrFE-CFE)/ HATCN/ H2:GD	Green	~17	6,601	120	1,000	Full cycle	NO	

	/Bphen/Liq/Al side-by-side ITO/PEDOT:PSS/P(VDF-TrFE-CFE)/ HATCN/ H2:GD /Bphen/Liq/Al side-by-side ITO/PEDOT:PSS/P(VDF-TrFE-CFE)/ HATCN/ H2:GD /Bphen/Liq/Al								
Multi electrodes	ITO/Au/Ag/Spiro-TTB:F6-TCNNQ/Spiro-TAD/O-EM/Bphen/Bphen:Cs/Au/Ag/Spiro-TTB:F6-TCNNQ/Spiro-TAD/O-EM/Bphen/Bphen:Cs/Au/Ag/α-NPD	White	9.7	N/A	N/A	50	Full cycle	NO	[26]
	ITO/Spiro-TTB:F6-TCNNQ/Spiro-TAD/α-NPD/4P-NPD/Bphen/$BAlq_3$/Bphen:Cs/Au/Ag/Spiro-TTB:F6-	White	36.8 (Yellow: 15-16% EQE; Blue:	N/A	N/A	50	Full cycle	NO	[27]

	TCNNQ/Spiro-TAD/TCTA:Ir(dhfpy)$_2$(acac)/TPBi:Ir(dhfpy)$_2$(acac)/BAlq$_3$/Bphen:Cs/Al			3.4% EQE)						
	ITO/PEDOT:PSS/TFB/QD/ZnO/ultra-thin Al/IZO/MoO$_3$/NPB/MADN:DSA-Ph/TPBi/LiF/Al		White	N/A	107,000	14.0	50	Full cycle	NO	[28]
	ITO/PEDOT:PSS/TFB/QD/ZnO/ultra-thin Al/IZO/MoO$_3$/NPB/MADN:DSA-Ph/TPBi/LiF/Al		Red	N/A	18,750	8.0	50	Full cycle	NO	[29]
	ITO/ZnO/QD/CBP/MoO$_3$/IZO/ZnO/QD/CBP/MoO$_3$/Al	AC-QLED	Red	23.16 (20.11% EQE)	15,700	6.0	50	Full cycle	NO	Our work
		PnP-QLED	Red	21.44 (37.05% EQE)	32,265	12.0	50	Full cycle	NO	
		(PnP-QLED)$_n$	Red	15.7	25,834	220	50	Full cycle	YES	

				(770% EQE)						

Supplementary Simulation Method

A classical formalism that describes the dipole radiation is used to model the light emission of the QLED devices. The method has been well documented by previous researchers [30–33]. In such a model, the emitter is considered to be a forced, damped, electric dipole oscillator located in a planar multilayer structure, as schematically shown in Supplementary Fig. 4a. The dipole is resided in region 1, and is sandwiched between a reflective region 2 and a transmissive region 3. The waves generated by the dipole radiation are reflected back and forth by the region 2 and the region 3. If the reflected waves are in phase, emission is enhanced and the exciton decay rate is increased. Otherwise, emission is inhibited and the exciton decay rate is reduced.

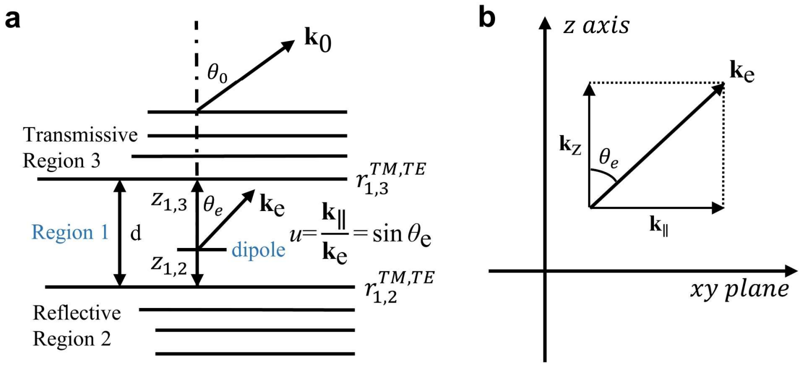

Supplementary Fig. 4 Optical model of a QLED device. a, A forced, damped, electric dipole oscillator located in a planar multilayer structure ($z_{1,2}$/ $z_{1,3}$ is the distances of the emitting dipoles from the bottom(between region 1 and 2)/top (between region 1 and 3) interfaces; $r_{1,2}^{\mathrm{TM,TE}}$/ $r_{1,3}^{\mathrm{TM,TE}}$ is the reflection coefficients of the bottom (between region 1 and 2)/top (between region 1 and 3) interface). TM/TE represents transverse magnetic/ transverse electric wave. **b,** The **k** vector of a plane wave can be decomposed to a vertical vector $\mathbf{k}_z$ and an in-plane vector $\mathbf{k}_{\|}$.

The dipole radiation is modeled as a sum of many plane waves. Each wave is characterized by a normalized in-plane wavevector u. As shown in Supplementary Fig. 4b, the wavevector $\mathbf{k}_e$ in the emission layer with a refractive index of n_e, can be

decomposed to a vertical wavevector $\mathbf{k}_{z,e}=\mathbf{k}_e\cos\theta_e$ and an in-plane wavevector $\mathbf{k}_{\|}=\mathbf{k}_e\sin\theta_e$, where θ_e is the angle between the $\mathbf{z}$ axis and the $\mathbf{k}_e$ vector. By definition, the normalized in-plane wavevector $u=\frac{\mathbf{k}_{\|}}{\mathbf{k}_e}=\sin\theta_e$, which also characterizes the emission angle of the dipole radiation. The vertical wavevector in layer i can then be deduced as:

$$k_{z,i}=\left(k_i^2-k_{\|}^2\right)^{1/2}=k_e\left(k_i^2/k_e^2-k_{\|}^2/k_e^2\right)^{1/2}=k_e\left(n_i^2/n_e^2-u^2\right)^{1/2} \tag{1}$$

where $k_e=n_e 2\pi/\lambda$ and n_i is the refractive index of i layer.

For the waves to propagate in the i layer, $k_{z,i}$ should be real, which means $u<n_i/n_e$. For example, for the waves to propagate in air, $u<1/n_e$. In the case that $k_{z,i}$ is complex, the waves are evanescent and decrease in amplitude along the z direction.

For the vertical dipole, the power intensity of the generated transverse magnetic (TM) wave at a wavelength of λ and a normalized in-plane wavevector u is:

$$K_{\mathrm{TMv}}=\frac{3}{2}\mathrm{Re}\left[\frac{u^3}{\sqrt{1-u^2}}\frac{\left(1+a_{1,2}^{\mathrm{TM}}\right)\left(1+a_{1,3}^{\mathrm{TM}}\right)}{1-a_{\mathrm{TM}}}\right] \tag{2}$$

For the horizontal dipole oriented in the plane, the power density of the generated TM and transverse electric (TE) waves at a wavelength of λ and a normalized in-plane wavevector u is:

$$K_{\mathrm{TMh}}=\frac{3}{4}\mathrm{Re}\left[u\sqrt{1-u^2}\frac{\left(1-a_{1,2}^{\mathrm{TM}}\right)\left(1-a_{1,3}^{\mathrm{TM}}\right)}{1-a_{\mathrm{TM}}}\right] \tag{3}$$

$$K_{\mathrm{TEh}}=\frac{3}{4}\mathrm{Re}\left[\frac{u}{\sqrt{1-u^2}}\frac{\left(1+a_{1,2}^{\mathrm{TE}}\right)\left(1+a_{1,3}^{\mathrm{TE}}\right)}{1-a_{\mathrm{TE}}}\right] \tag{4}$$

where Re[…] represents the real part of the complex quantity enclosed by brackets. Note that in some papers[30,31] the K denotes the power density per unit $\mathrm{d}u^2$, and in that case, Eqs. (2)-(4) should be divided by $2u$. Also in some papers[32,33], the signs before the $a_{1,2}^{\mathrm{TM}}$ and $a_{1,3}^{\mathrm{TM}}$ are opposite to ours, and this is because we chose a different vector direction when deriving the TM reflection coefficients, as will be discussed later.

In the Eqs. (2)-(4), the factor $1\pm a_{12,13}$ in the numerator describes the wide-angle interference between directly emitted and reflected radiation. The factor $1-a_{\mathrm{TM,TE}}$ in

the denominator describes the multiple-beam interference that occurs when the waves are reflected back and forth between region 2 and region 3. Furthermore,

$$a_{1,2}^{\mathrm{TM,TE}}=r_{1,2}^{\mathrm{TM,TE}}\exp\left(2jk_{\mathrm{z,e}}z_{1,2}\right) \tag{5}$$

$$a_{1,3}^{\mathrm{TM,TE}}=r_{1,3}^{\mathrm{TM,TE}}\exp\left(2jk_{\mathrm{z,e}}z_{1,3}\right) \tag{6}$$

$$a_{\mathrm{TM,TE}}=a_{1,2}^{\mathrm{TM,TE}}a_{1,3}^{\mathrm{TM,TE}}=r_{1,2}^{\mathrm{TM,TE}}r_{1,3}^{\mathrm{TM,TE}}*\exp\left(2jk_{\mathrm{z,e}}d\right) \tag{7}$$

where $r_{1,2}^{\mathrm{TM,TE}}$ and $r_{1,3}^{\mathrm{TM,TE}}$ are the reflection coefficients of the bottom interface (between region 1 and 2) and the top interface (between region 1 and 3), respectively. $z_{1,2}$ and $z_{1,3}$ are the distances of the emitting dipoles from the bottom and the top interfaces, respectively, and d is the thickness of the emitting layer.

For the multiplayer structure with many interfaces, the reflection at each interface should be taken into account. The complex Fresnel reflection coefficients of the interface between i and i+1 layer are

$$r_{i,i+1}^{\mathrm{TM}}=\frac{n_{i+1}^2k_{\mathrm{z},i}-n_i^2k_{\mathrm{z},i+1}}{n_{i+1}^2k_{\mathrm{z},i}+n_i^2k_{\mathrm{z},i+1}} \tag{8}$$

$$r_{i,i+1}^{\mathrm{TE}}=\frac{k_{\mathrm{z},i}-k_{\mathrm{z},i+1}}{k_{\mathrm{z},i}+k_{\mathrm{z},i+1}} \tag{9}$$

Note that the sign of the reflection coefficient in Eq. (8) is different with that reported in other papers or textbooks[32,33]. They differ because in this paper, the direction of the electric field (**E**) vector (Supplementary Fig. 5) of the reflective waves for the TM polarization is opposite to those in most reports[32,33]. Keeping such difference in mind when evaluating the quantities relating to the TM reflection coefficients [for example, Eqs. (2), (3), (5), (6)], the same results can be obtained.

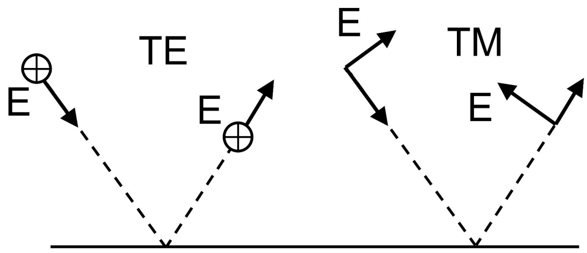

Supplementary Fig. 5 The direction of the electric field vector. Sign convention for reflection coefficients in the case for TE and TM polarization. Note that the direction of E vector of the reflective waves of the TM polarization is opposite to those in most reports.

For multiple layers, starting from the outermost layer, the total reflection coefficients of all layers can then be obtained by iteratively calculating

$$r_{i,j}^{\mathrm{TM,TE}}=\frac{r_{i,i+1}^{\mathrm{TM,TE}}+r_{i+1,j}^{\mathrm{TM,TE}}\exp\left(2jk_{z,i+1}d_{i+1}\right)}{1+r_{i,i+1}^{\mathrm{TM,TE}}\mathrm{r}_{i+1,j}^{\mathrm{TM,TE}}\exp\left(2jk_{z,i+1}d_{i+1}\right)} \tag{10}$$

For an isotropic dipole distribution, the total spectral power K per unit normalized in-plane wave vector is finally obtained as

$$K=\frac{1}{3}\left(K_{\mathrm{TMv}}+2K_{\mathrm{TMh}}+2K_{\mathrm{TEh}}\right) \tag{11}$$

The total radiated power by the dipole emitter at λ can be obtained by integrating the area under the power dissipation spectrum.

$$F(\lambda)=\int_0^{\infty}K(\lambda,u)\mathrm{d}u \tag{12}$$

The F is also called as the Purcell factor, which is determined by the device structure. Additionally, we can compute the fractional power coupled to a specific mode by integrating the area under the power dissipation spectrum corresponding to that mode, and then divided it by the total power.

The far field radiation which determines the useful light emission can be calculated by taking the transmittance of the top surface (region 3) into account. For example, the outcoupled power fractions K'_{TMv}, K'_{TMh}, K'_{TEh}, and K' radiated into the substrate for $u<n_{\mathrm{sub}}/n_{\mathrm{e}}$ are calculated as

$$K'_{\mathrm{TMv}}=\frac{3}{4}\frac{u^3}{\sqrt{1-u^2}}\frac{|1+a_{1,2}^{\mathrm{TM}}|^2}{|1-a_{\mathrm{TM}}|^2}T_{1,3}^{\mathrm{TM}} \tag{13}$$

$$K'_{\mathrm{TMh}}=\frac{3}{8}u\sqrt{1-u^2}\frac{|1-a_{1,2}^{\mathrm{TM}}|^2}{|1-a_{\mathrm{TM}}|^2}T_{1,3}^{\mathrm{TM}} \tag{14}$$

$$K'_{\mathrm{TEh}}=\frac{3}{8}\frac{u}{\sqrt{1-u^2}}\frac{|1+a_{1,2}^{\mathrm{TE}}|^2}{|1-a_{\mathrm{TE}}|^2}T_{1,3}^{\mathrm{TE}} \tag{15}$$

$$K'=\frac{1}{3}\left(K'_{\mathrm{TMv}}+2K'_{\mathrm{TMh}}+2K'_{\mathrm{TEh}}\right) \tag{16}$$

where $T_{1,3}^{\mathrm{TM,TE}}$ denote the energy transmittance of the top surface (region 3) and can be calculated by

$$T_{1,3}^{\mathrm{TM}}=|t_{1,3}^{\mathrm{TM}}|^2\frac{k_{\mathrm{z,sub}}}{k_{\mathrm{z,e}}} \tag{17}$$

$$T_{1,3}^{\mathrm{TE}}=|t_{1,3}^{\mathrm{TE}}|^2\frac{k_{\mathrm{z,sub}}}{k_{\mathrm{z,e}}} \tag{18}$$

The transmittance coefficients $t_{1,3}^{\mathrm{TM,TE}}$ of the top surface (region 3) can be calculated by using the transfer matrix method.

The fractional power coupled into the air for $u<1/n_{\mathrm{e}}$ is finally obtained by considering the reflectance R_{s} and the transmittance T_{s} of the interface between glass substrate and air.

$$K_{\mathrm{out}}=K'(u<1/n_{\mathrm{e}})\frac{T_{\mathrm{s}}}{1-R_{\mathrm{s}}R_{\mathrm{c}}} \tag{19}$$

where R_{c} is the total reflectance of all device layers.

The total power coupled into the air can then obtained by integrating K_{out} over all directions in the light escape cone:

$$U(\lambda)=\int_0^{\frac{1}{n_{\mathrm{e}}}}K_{\mathrm{out}}(\lambda,u)\mathrm{d}u \tag{20}$$

The coupling efficiency can be obtained by

$$\eta_{\mathrm{out}}(\lambda)=\frac{U(\lambda)}{F(\lambda)} \tag{21}$$

At the presence of cavity structure, the exciton radiated decay rate will be modified by the Purcell factor and thus the quantum yield (QY) of the emitter is also modified as

$$\eta_{\text{rad, cav}}(\lambda)=\frac{Fk_{\text{r}}}{Fk_{\text{r}}+k_{n\text{r}}}=\frac{Fk_{\text{r}}/(k_{\text{r}}+k_{n\text{r}})}{(Fk_{\text{r}}+k_{n\text{r}})/(k_{\text{r}}+k_{n\text{r}})}=\frac{F\eta_{\text{rad}}}{1-\eta_{\text{rad}}+F\eta_{\text{rad}}} \tag{22}$$

where k_{r} and $k_{n\text{r}}$ are the radiated and the non-radiated decay rates, respectively, and $\eta_{\text{rad}}=k_{\text{r}}/(k_{\text{r}}+k_{n\text{r}})$ is the intrinsic emitter quantum yield.

The QY enhancement due to the presence of cavity thus is

$$\eta_{\text{QY,enhancement}}=\frac{\eta_{\text{rad,cav}}}{\eta_{\text{rad}}}==\frac{F}{1-\eta_{\text{rad}}+F\eta_{\text{rad}}} \tag{23}$$

In the case that the η_{rad} is low, which means the $k_{n\text{r}}$ is the dominant transition, the QY of the emitter can be significantly improved since the k_{r} can be effectively accelerated by the cavity. In the case that the η_{rad} is relatively high, meaning $k_{\text{r}}\gg k_{n\text{r}}$, the QY enhancement through speedy the k_{r} is marginal.

Considering the modification of the cavity structure on both coupling efficiency and QY of the emitter, the final improvement due to the cavity thus is

$$\eta_{\text{enhancement}}(\lambda)=\frac{\eta_{\text{rad,cav}}}{\eta_{\text{rad}}}\eta_{\text{out}}=\frac{1}{1-\eta_{\text{rad}}+F\eta_{\text{rad}}}\int_{0}^{\frac{1}{n_{\text{e}}}}K_{\text{out}}(\lambda,u)\text{d}u \tag{24}$$

which normally is larger than η_{out} due to the contribution of higher QY induced by the cavity. For emitter with a lower QY, the $\eta_{\text{enhancement}}$ could be significantly higher than η_{out}. It is more accurate to evaluate the light enhancement efficiency of the structure by using Eq. (24).

The external quantum efficiency (EQE) of the device can be obtained by introducing the charge balance efficiency γ

$$\eta_{\text{EQE}}=\gamma\eta_{\text{rad,cav}}\eta_{\text{out}}=\gamma\int_{\lambda_{\text{min}}}^{\lambda_{\text{max}}}s_{\text{pl}}(\lambda)\frac{\eta_{\text{rad}}}{1-\eta_{\text{rad}}+F\eta_{\text{rad}}}U(\lambda)\text{d}\lambda \tag{25}$$

where $s_{\text{pl}}(\lambda)$ is the normalized photoluminescence (PL) spectrum of the device which satisfies $\int_{\lambda_{\text{min}}}^{\lambda_{\text{max}}}s_{\text{pl}}(\lambda)\text{d}\lambda=1$. At low level excitation such that the Auger recombination and Joule heat activated non-radiated recombination could be neglected, the γ can be extracted by fitting the measured EQE with the calculated η_{EQE}.

The above spectral power quantities per unit normalized in-plane wavevector can be converted into the power densities per unit solid angle by using the relation:

$$\text{total far field radiated power}=\int_0^{\arcsin\left(\frac{1}{n_e}\right)} 2\pi P_{out}(\lambda,\theta)\sin\theta d\theta=\int_0^{\frac{1}{n_e}} K_{out}(\lambda,u)du \tag{26}$$

In the above equation, the integral range for the left and the right integrations is the same, and thus the integral element for the left and the right integrations is also the same.

By using the Fresnel relation

$$n_0 \sin(\theta) = n_e \sin\theta_e = n_e u \tag{27}$$

have $u=\frac{n_0}{n_e}\sin(\theta)=\frac{\sin(\theta)}{n_e}$ and thus $\frac{du}{d\theta}=\frac{\cos(\theta)}{n_e}$.

Finally we can deduce

$$P_{out}(\lambda,\theta)=\frac{1}{2\pi}\frac{1}{n_e}\frac{\cos(\theta)}{\sin(\theta)}K_{out}(\lambda,u) \tag{28}$$

where $\theta=\arcsin(n_e u)$ is the light emitting angle in air. The angularly dependent spectrum of the device can then be obtained by

$$S(\lambda,\theta)=s_{pl}(\lambda)P_{out}(\lambda,\theta) \tag{29}$$

By integrating the spectrum density over all wavelength, the angularly dependent emission intensity can be obtained as

$$I(\theta)=\int_{\lambda_{min}}^{\lambda_{max}} s_{pl}(\lambda)\, P_{out}(\lambda,\theta)d\lambda \tag{30}$$

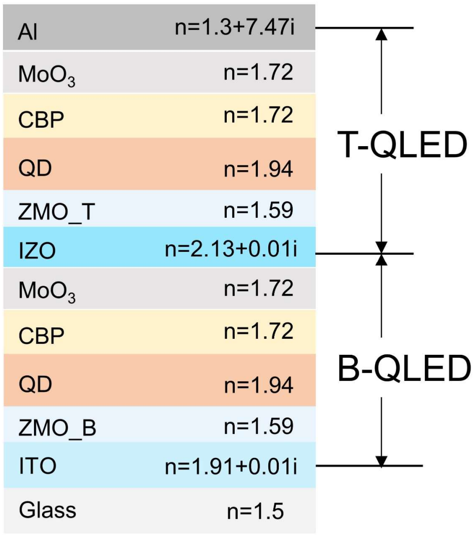

Supplementary Fig. 6 Optical properties of the functional layers. The schematic device structure of B-QLED and T-QLED and the parameters for simulation at a wavelength of 626 nm. B-QLED/T-QLED represents the bottom/top QLED in tandem QLED. ZMO_B/ZMO_T represents the bottom/top ZnMgO layer. The quantum yield of the red QD emitter is 89.97%.

Based on the aforementioned simulation process, we can simulate the device's light outcoupling efficiencies (OCEs). The refractive index of each layer used for simulation are shown in Supplementary Fig. 6.

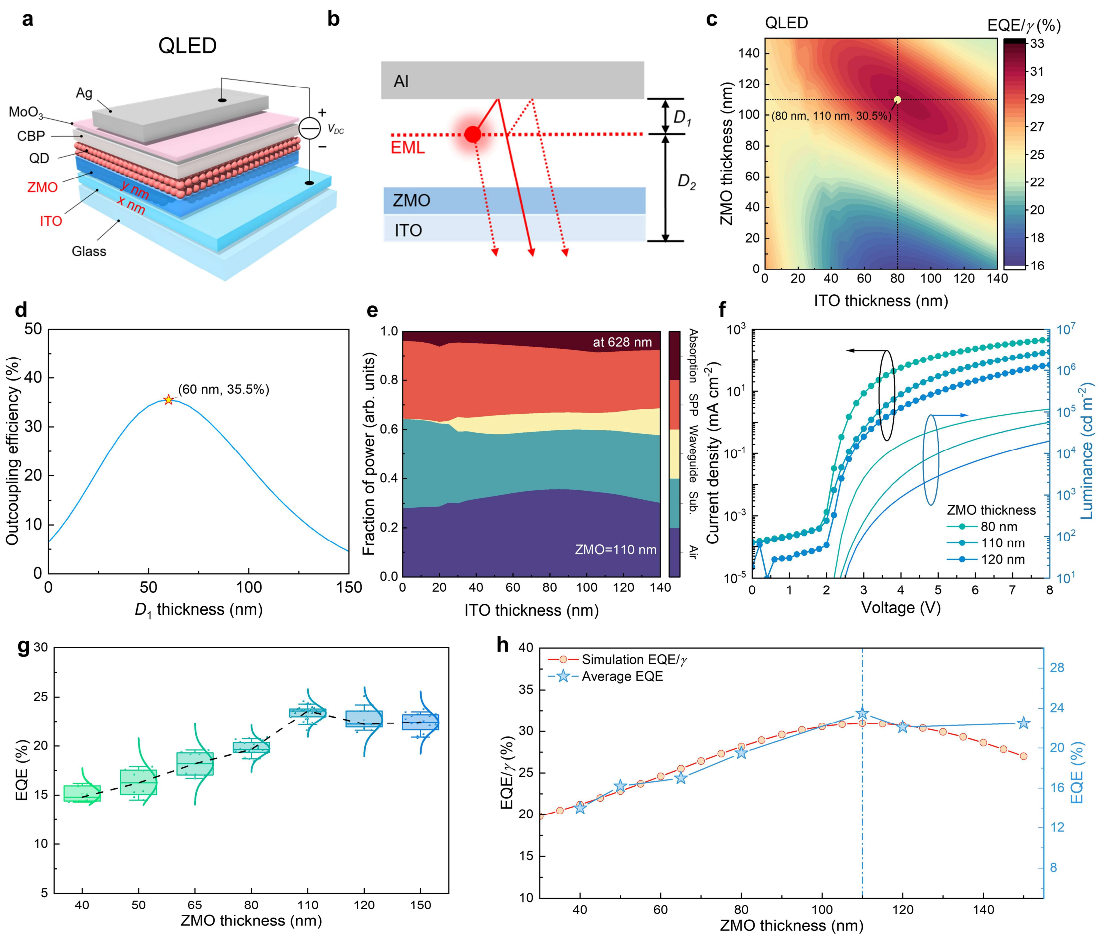

Supplementary Fig. 7 Simulated results and performance of single QLED. a, Device structure (glass/ITO *x* nm/ZMO *y* nm/QD 20 nm/CBP 50 nm/MoO_3 10 nm/Al 100 nm). ZMO represents the ZnMgO layer. **b**, Schematic diagram of wide-angle interference in QLED. EML represents emission layer. D_1 and D_2 are the distances of the recombination zone from the top Al electrode and the bottom ITO electrode, respectively. **c,** Outcoupling efficiencies (OCEs) of QLED as a function of the thickness of ZMO and ITO (γ: charge balance efficiency). **d**, the OCEs of QLED as a function of the thickness of D_1 (CBP and MoO_3). Our previous work[34] indicates that a QLED, at the optimal resonant cavity (D_1) thickness of 60 nm, can exhibit the highest performance, confirming the accuracy of the optical simulations. Therefore, during the optimization process of the OCEs of the stacked device, the D_1 (CBP and MoO_3) was fixed at 60 nm. **e**, Power fraction of each mode as a function of the ITO thickness in red QLED with 110 nm ZMO. **f**, *J-V-L* characteristic curves of the QLEDs with different thickness of ZMO. **g**, Maximum EQE statistics of the QLEDs with different

ZMO thickness. **h**, Comparison of experimental results (blue star) and simulation results (red circle) for EQE of QLED as a function of ZMO thickness.

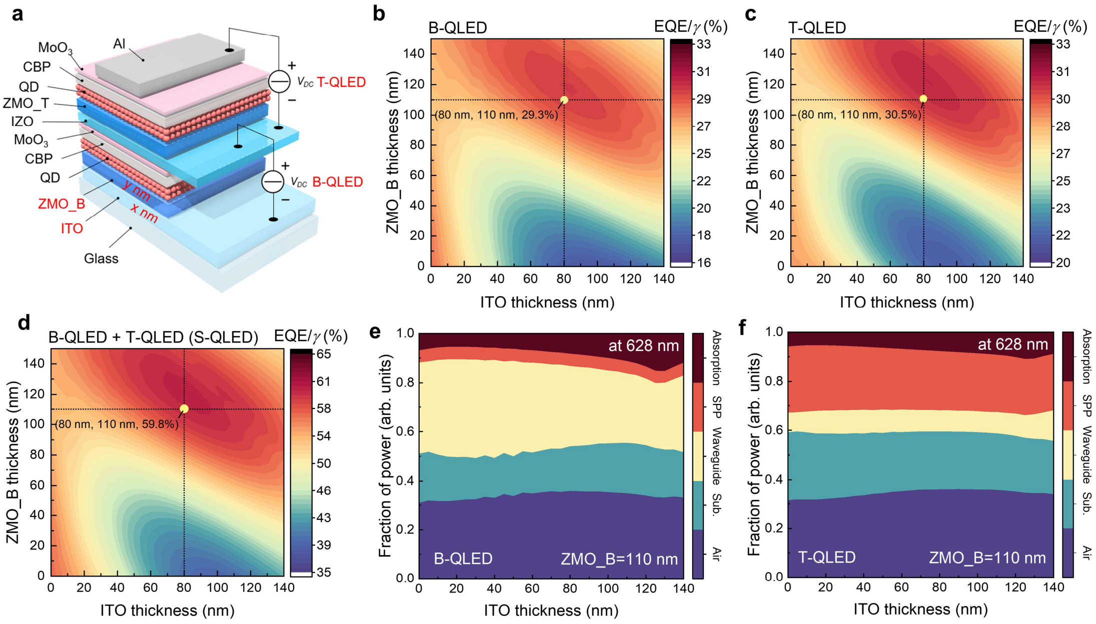

Supplementary Fig. 8 Optical simulation results of tandem QLED. a, Device structure (glass/ITO *x* nm/ZMO_B *y* nm/QD 20 nm/CBP 50 nm/MoO_3 110 nm/IZO 30 nm/ZMO_T 80 nm/QD 20 nm/CBP 50 nm/MoO_3 10 nm/Al 100 nm). B-QLED/T-QLED represents the bottom/top QLED in tandem QLED. ZMO_B/ZMO_T represents the bottom/top ZnMgO layer**. b-d**, Simulated outcoupling efficiencies (OCEs) of B-QLED (**b**), T-QLED (**c**), and S-QLED (**d**), respectively, as a function of the thickness of ZMO_B and ITO (γ: charge balance efficiency). **e**, **f**, Power fraction of each mode of B-QLED (**e**), and T-QLED (**f**), with 110 nm ZMO_B, respectively, as a function of the ITO thickness.

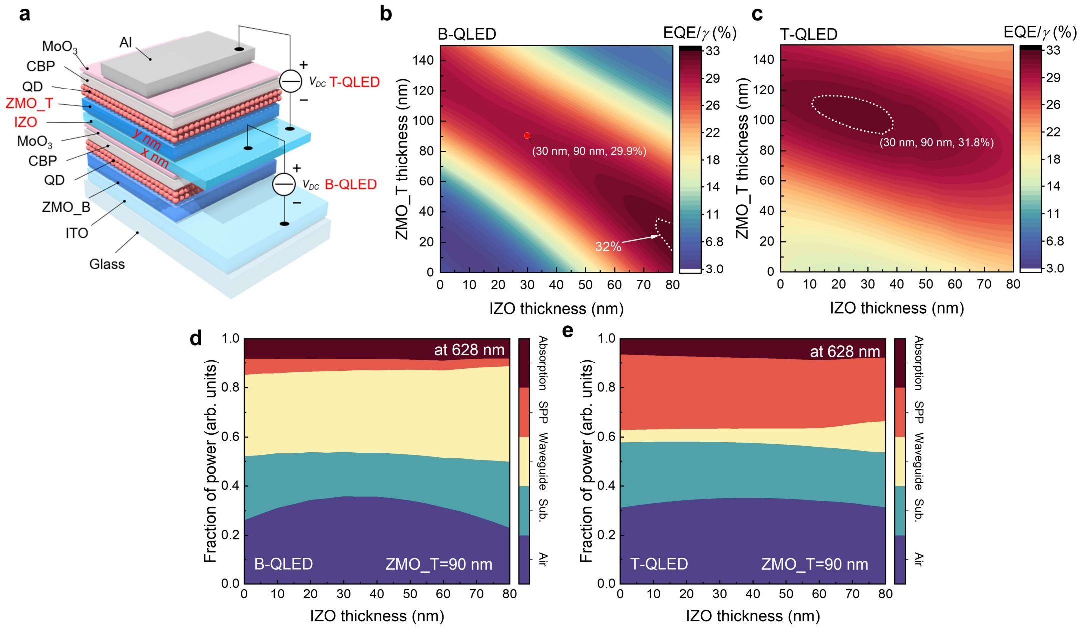

Supplementary Fig. 9 Optical simulation results of tandem QLED. a, Device structure (glass/ITO 80 nm/ZMO_B 110 nm/QD 20 nm/CBP 50 nm/MoO_3 10 nm/IZO x nm/ZMO_T y nm/QD 20 nm/CBP 50 nm/MoO_3 10 nm/Al 100 nm). B-QLED/T-QLED represents the bottom/top QLED in tandem QLED. ZMO_B/ZMO_T represents the bottom/top ZnMgO layer. **b**, **c**, Simulated outcoupling efficiencies (OCEs) of B-QLED (**b**), and T-QLED (**c**), respectively, as a function of the thickness of ZMO_T and IZO (γ: charge balance efficiency). **d**, **e**, Power fraction of each mode of B-QLED (**d**), and T-QLED (**e**), with 110 nm ZMO_T, respectively, as a function of the IZO thickness.

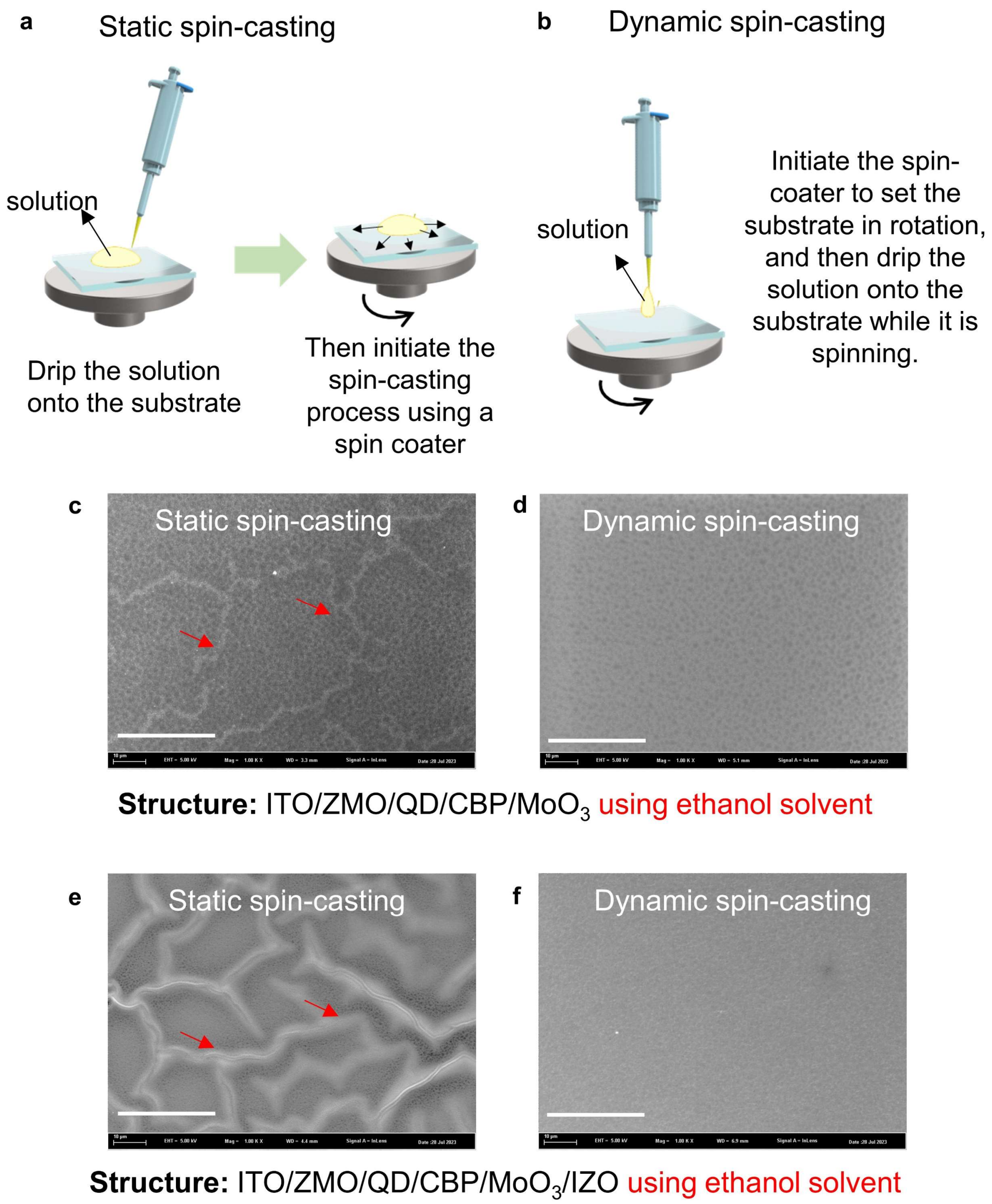

Supplementary Fig. 10 The impact of different spin-coating processes on the thin film. a, **b,** Schematic illustration of the static spin-casting (**a**), and the dynamic spin-casting processes (**b**), respectively. **c**, **d**, SEM images of sample (glass/ITO/ZMO/QD/CBP/MoO_3) surface after static spin-casting (**c**), and dynamic spin-casting (**d**), using ethanol solvent, respectively. Scale bars, 30 μm. **e**, **f**, SEM images of sample (glass/ITO/ZMO/QD/CBP/MoO_3/IZO) surface after static spin-casting (**e**), and dynamic spin-casting (**f**), using ethanol solvent, respectively. Scale bars, 30 μm.

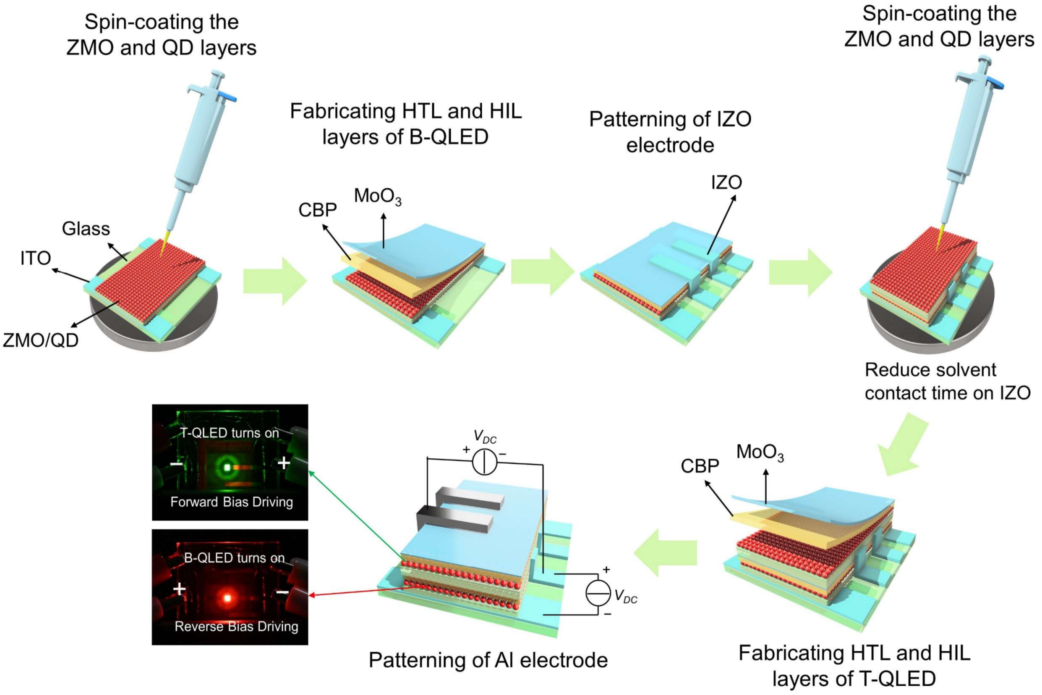

Supplementary Fig. 11 Fabrication processes of the tandem QLED. Schematics of the fabrication process for producing damage-free tandem QLEDs, resulting in uniform light emission from both B-QLED and T-QLED, as well as the tandem QLED. B-QLED/T-QLED represents the bottom/top QLED in tandem QLED.

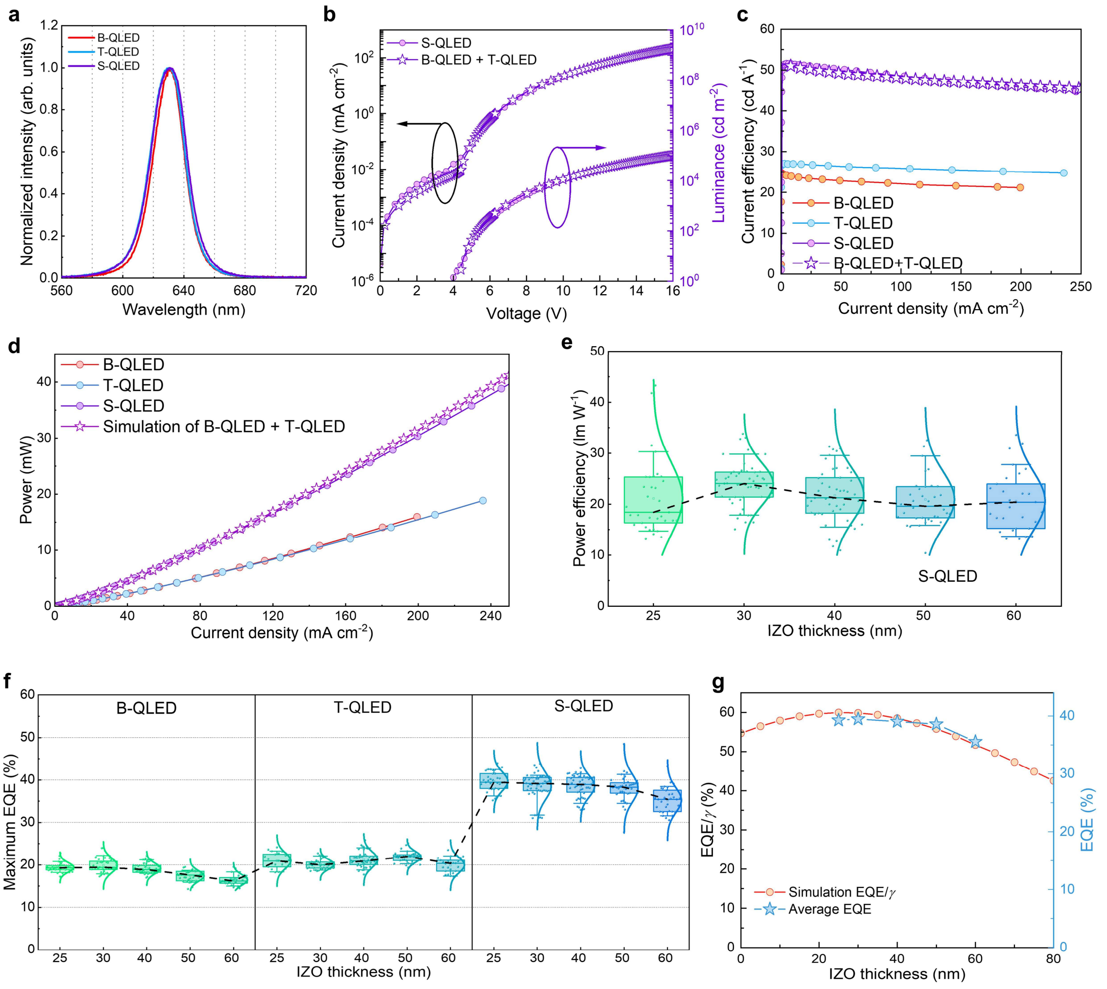

Supplementary Fig. 12 Optimization of red tandem QLED. a, The normalized electroluminescence (EL) spectra of B-QLED (red), T-QLED (blue), and S-QLED (purple). B-QLED/T-QLED represents the bottom/top QLED in tandem QLED. **b-d**, The *J-V-L* (**b**), CE-*J* (**c**), and *P-J* characteristics (**d**), of B-QLED, T-QLED and S-QLED, respectively: the current, current efficiency (CE) and power (*P*) of the S-QLED (purple solid circle) are perfectly equal to the summation of those of B-QLED and T-QLED (purple open star). **e**, Maximum power efficiency (PE) statistics of the S-QLEDs with different IZO thickness. **f**, Maximum EQE statistics of the B-QLEDs, T-QLEDs, and S-QLEDs with different IZO thickness. **g**, Comparison of experimental results (blue star) and simulation results (red circle) for EQE of S-QLED as a function of IZO thickness.

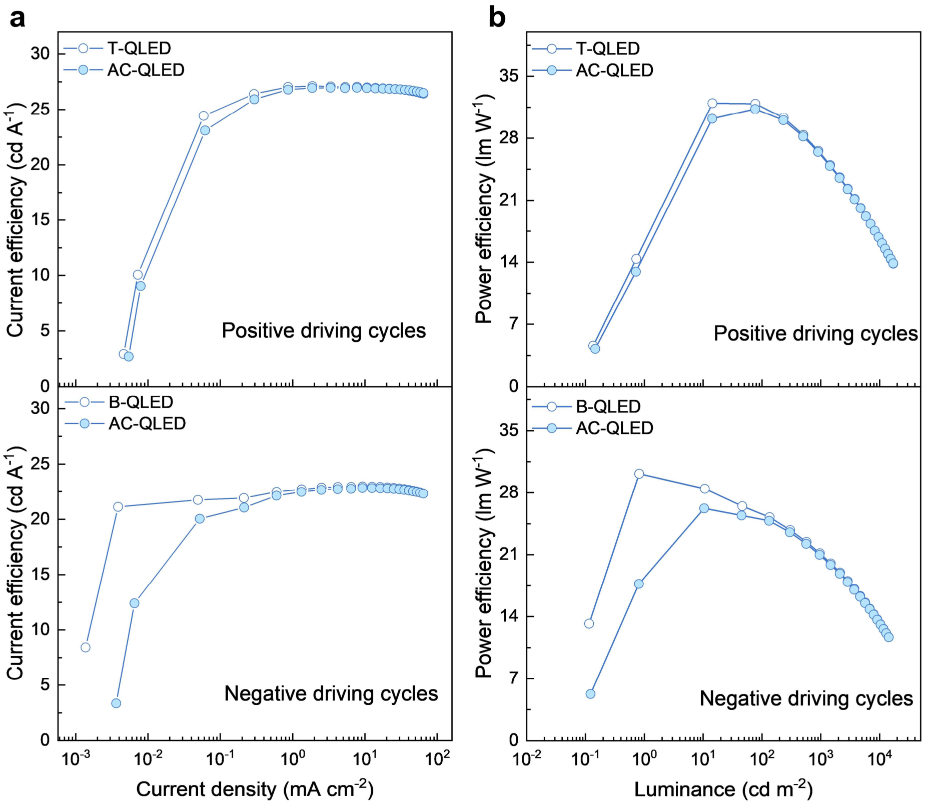

Supplementary Fig. 13 Performance of AC-QLED. The CE-*J* **a**, and PE-*L* **b**, characteristic curves of devices under positive or negative driving cycles, respectively.

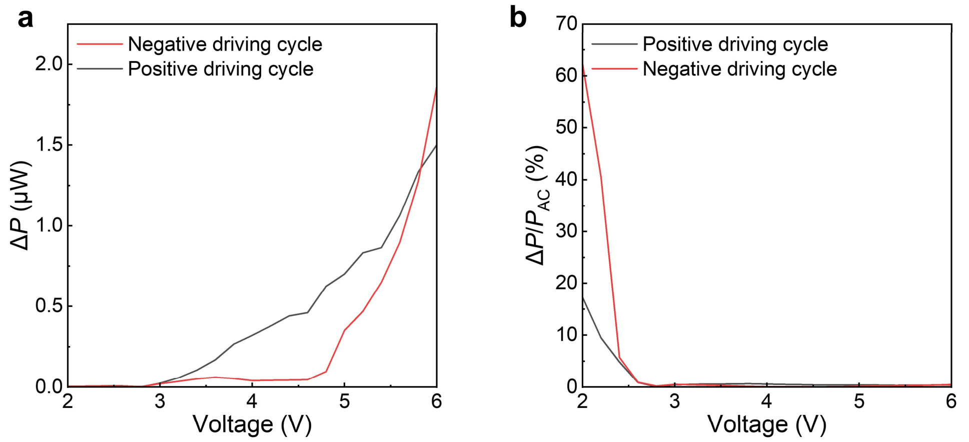

Supplementary Fig. 14 AC-QLED energy loss under different AC driving cycles. The energy loss of AC-QLED is presented during positive and negative driving cycles and can be calculated by $\Delta P = P_{AC} - P_S$, where P_{AC} is the power of AC-QLED during positive or negative driving cycles and P_S is the power of a single QLED (T-QLED or B-QLED). The ΔP-V **a**, and $\Delta P/P_{AC}$-V characteristic curves **b**, of AC-QLED under positive or negative driving cycles, respectively.

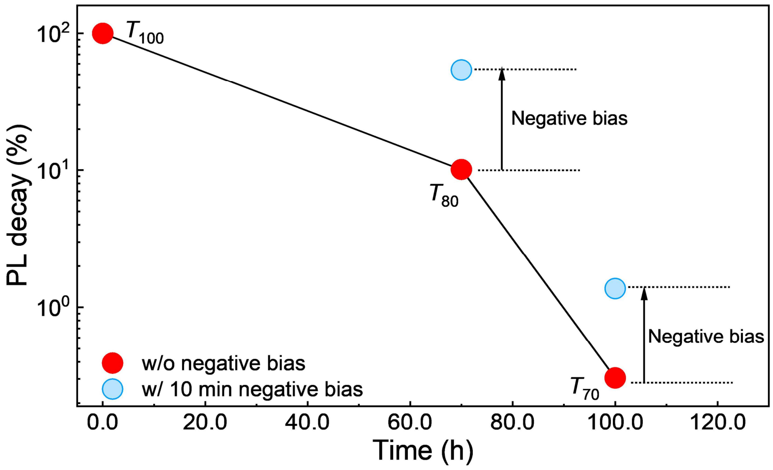

Supplementary Fig. 15 The decay of PL of QLED at different aging time. After applying a negative voltage for 10 min, the accumulated charges can be effectively released, and thus the photoluminescence (PL) of red QDs in QLED can be partially recovered.

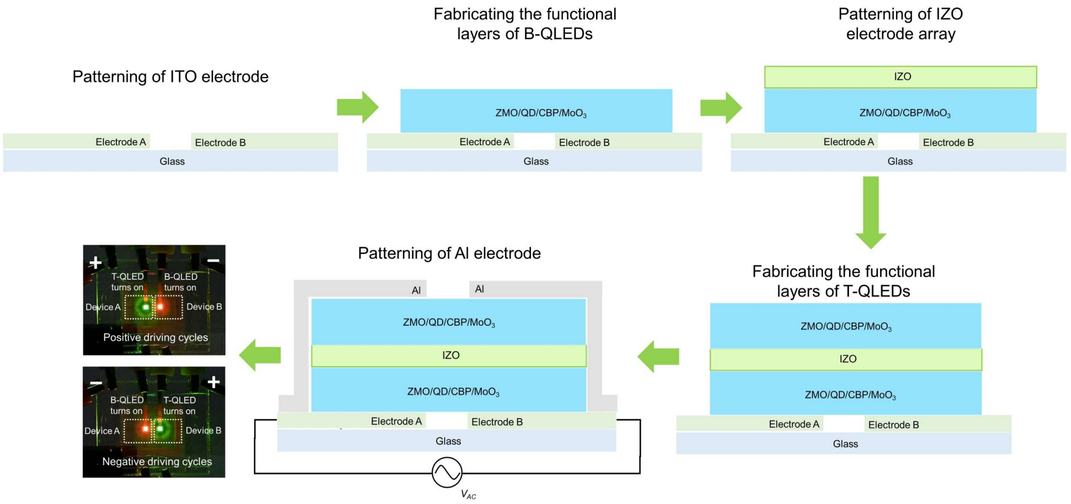

Supplementary Fig. 16 Schematic fabrication processes of a PnP-QLED. The diagrams portray the fabrication steps for creating plug-and-play QLEDs, which include two AC-QLEDs placed adjacent to each other and linked in series through the intermediary IZO electrode. Regardless of the driving cycles, light emission consistently emanates from both a T-QLED and a B-QLED. This structure ensures that the PnP-QLED maintains a uniform brightness throughout the entire AC operation.

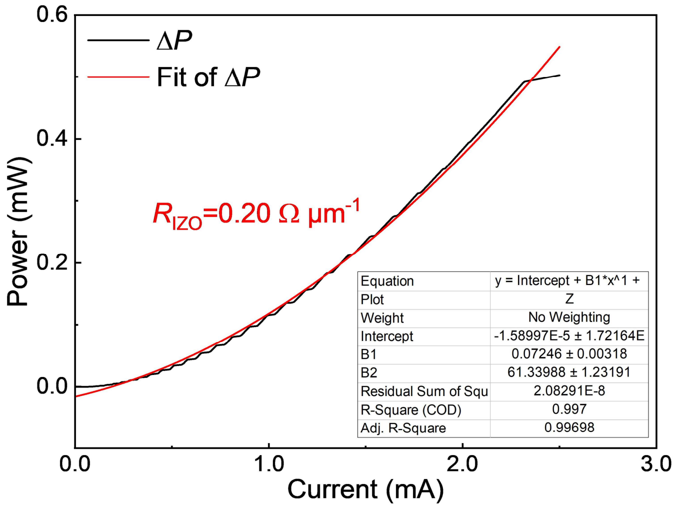

Supplementary Fig.17 Resistance of IZO wire. *P-I* characteristic curves of Δ*P* between two PnP-QLEDs with 0.2 mm and 0.5 mm IZO wire, where *P* is the power of PnP-QLED and Δ*P* is power difference between the PnP-QLEDs with a 0.2 mm and a 0.5 mm long IZO wire. By fitting curve using $\Delta P = I^2 R_{IZO}$, where *I* is the current in the PnP-QLED, the resistance of IZO wire (R_{IZO}) can be calculated as 0.2 Ω μm^{-1}.

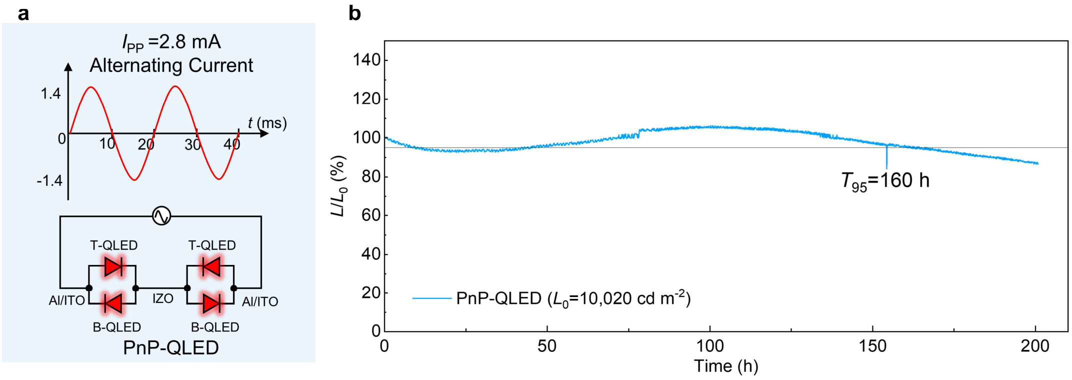

Supplementary Fig.18 Lifetime of PnP-QLED driven by alternating current. a, The driving conditions for plug-and-play QLED (PnP-QLED) for lifetime testing. The PnP-QLED is powered at 50 Hz and 2.8 mA peak-to-peak sinusoidal alternating current (I_{PP}). **b**, The lifetime curves of the PnP-QLED at an initial brightness of 10,020 cd m^{-2}.

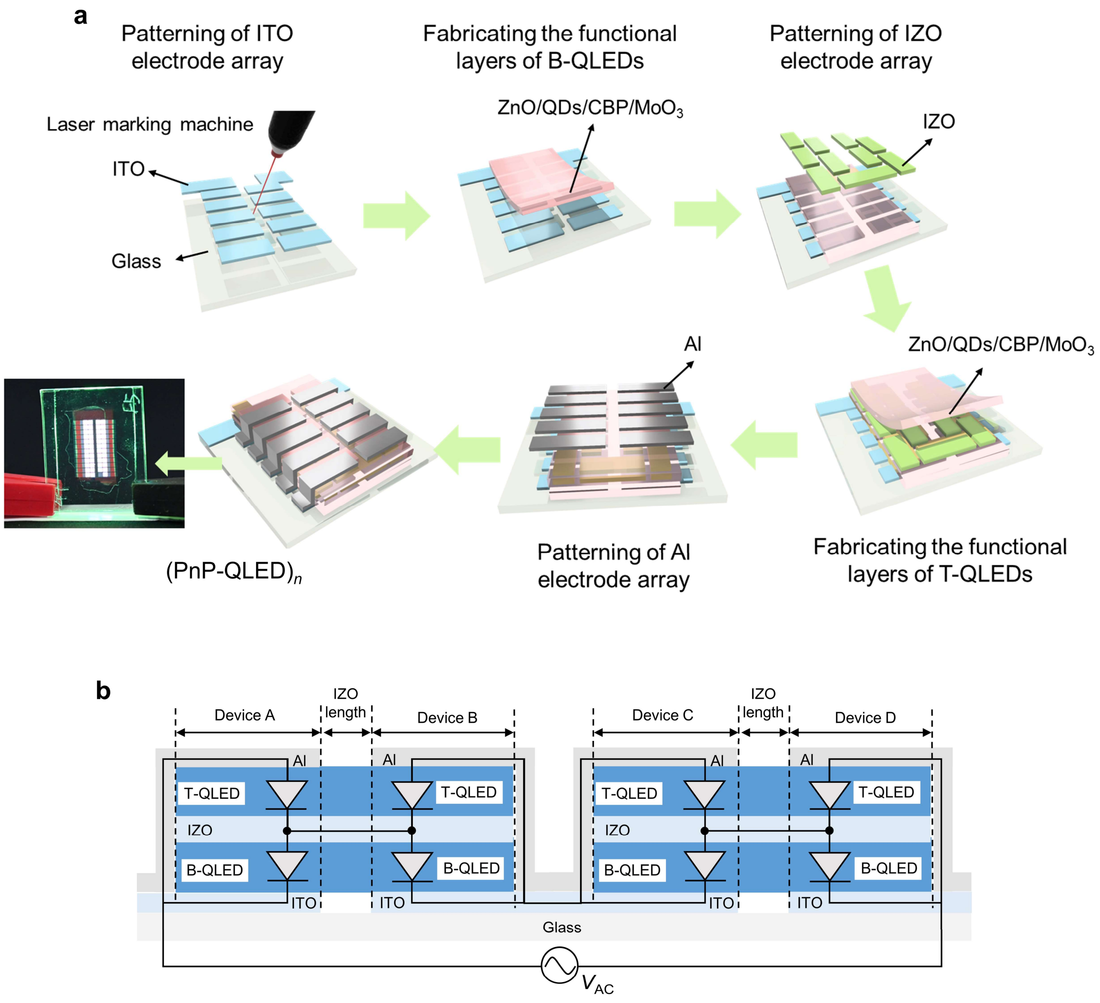

Supplementary Fig. 19 The structure and fabrication procedure of (PnP-QLED)*n*.

a, Fabrication processes of the (PnP-QLED)*n*. **b**, Schematic device structure of a (PnP-QLED)*n*. The PnP-QLEDs are connected by Al wires, which are highly conductive and thus the power consumption caused by Al wires is negligible.

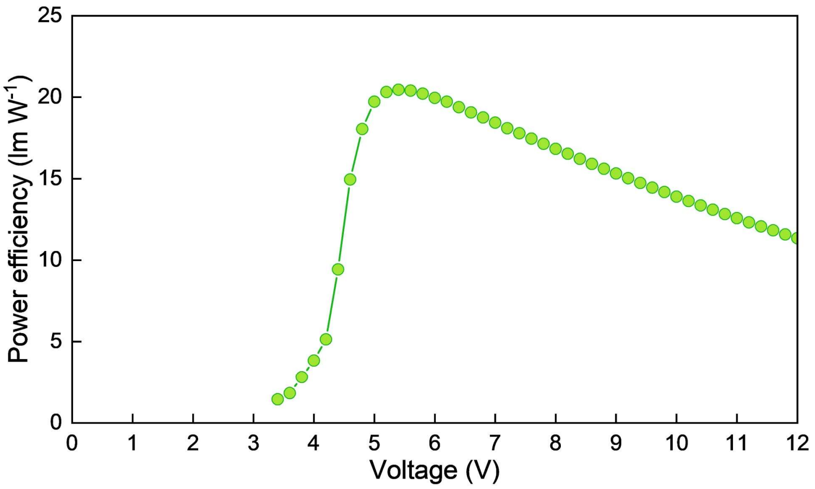

Supplementary Fig. 20 Performance of PnP-QLED. PE-*V* curve of a basic PnP-QLED.

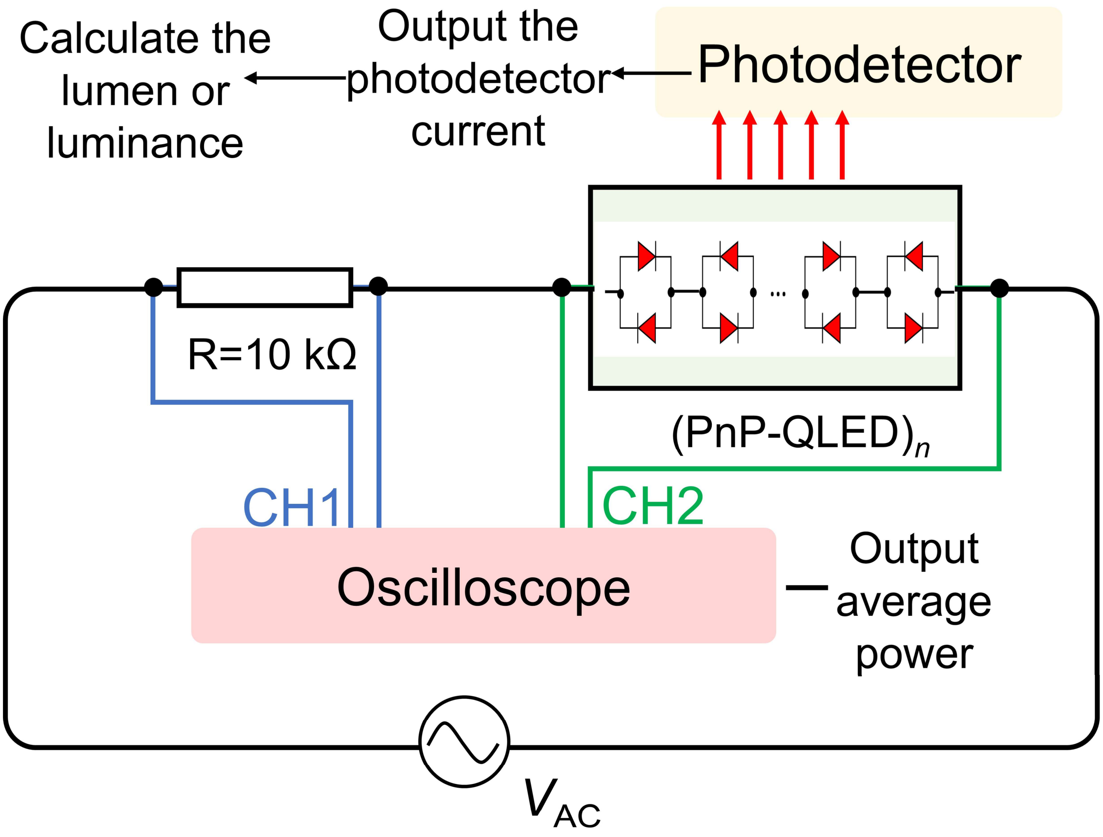

Supplementary Fig. 21 The performance measurement setup for (PnP-QLED)$_n$. The (PnP-QLED)$_n$ were driven by a AC electricity at 50 Hz using an isolation transformer. The AC voltage dropped across the (PnP-QLED)$_n$ and the current flowing through the (PnP-QLED)$_n$ were recorded by a dual-channel oscilloscope (Tektronix, TBS1102). The luminous flux generated by the (PnP-QLED)$_n$ was measured by a PIN-25D calibrated silicon photodiode.

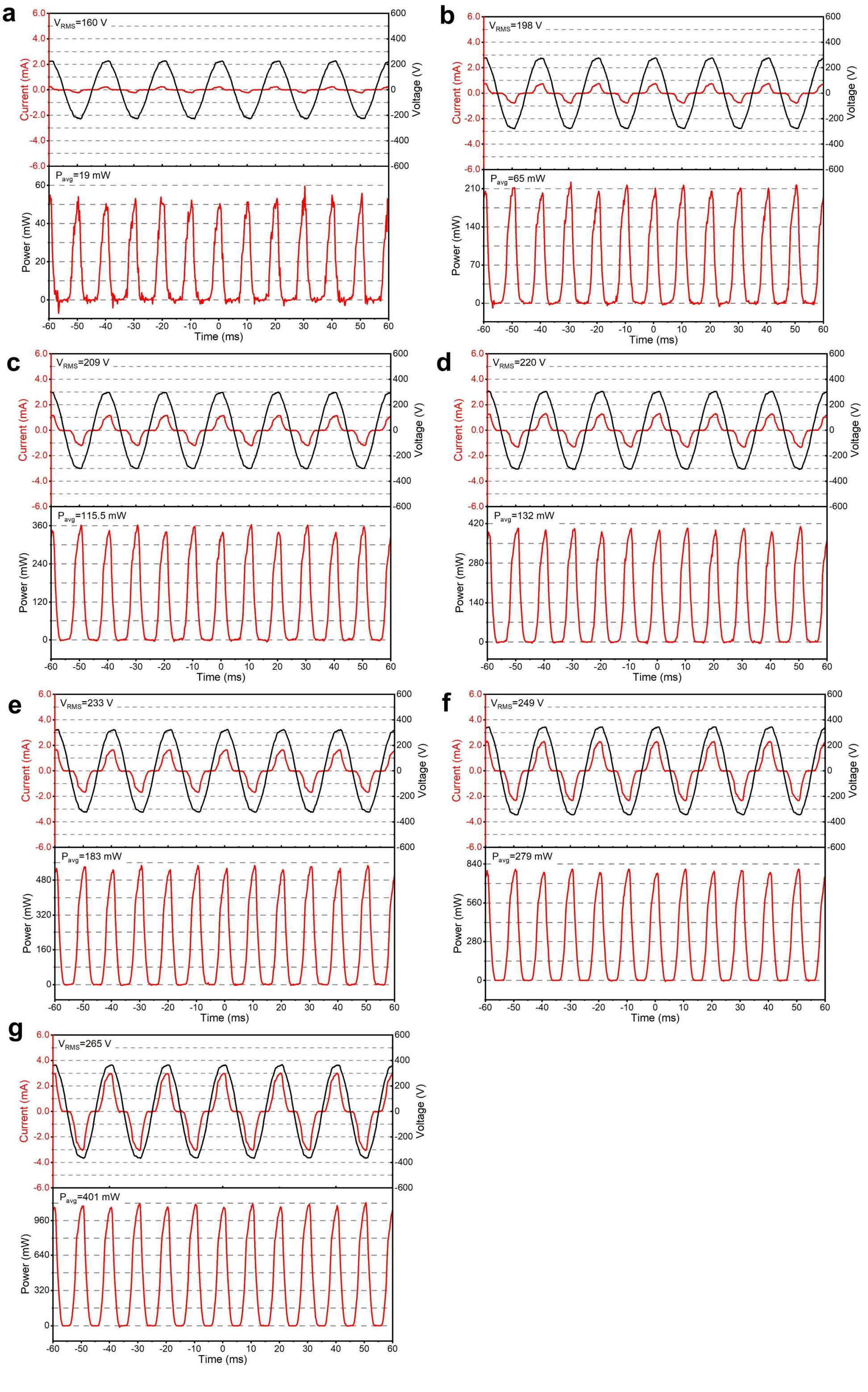

Supplementary Fig. 22 (PnP-QLED)$_{30}$ performance. a-g, *J*-*T*-*V* characterization and *P*-*T* characterization at V_{RMS}= 160 V (P_{avg}=19 mW) (**a**), 198 V (P_{avg}=65 mW) (**b**), 209 V (P_{avg}=115.5 mW) (**c**), 220 V (P_{avg}=132 mW) (**d**), 233 V (P_{avg}=183 mW) (**e**), 249 V (P_{avg}=279 mW) (**f**), 265 V (P_{avg}=401 mW) (**g**), respectively.

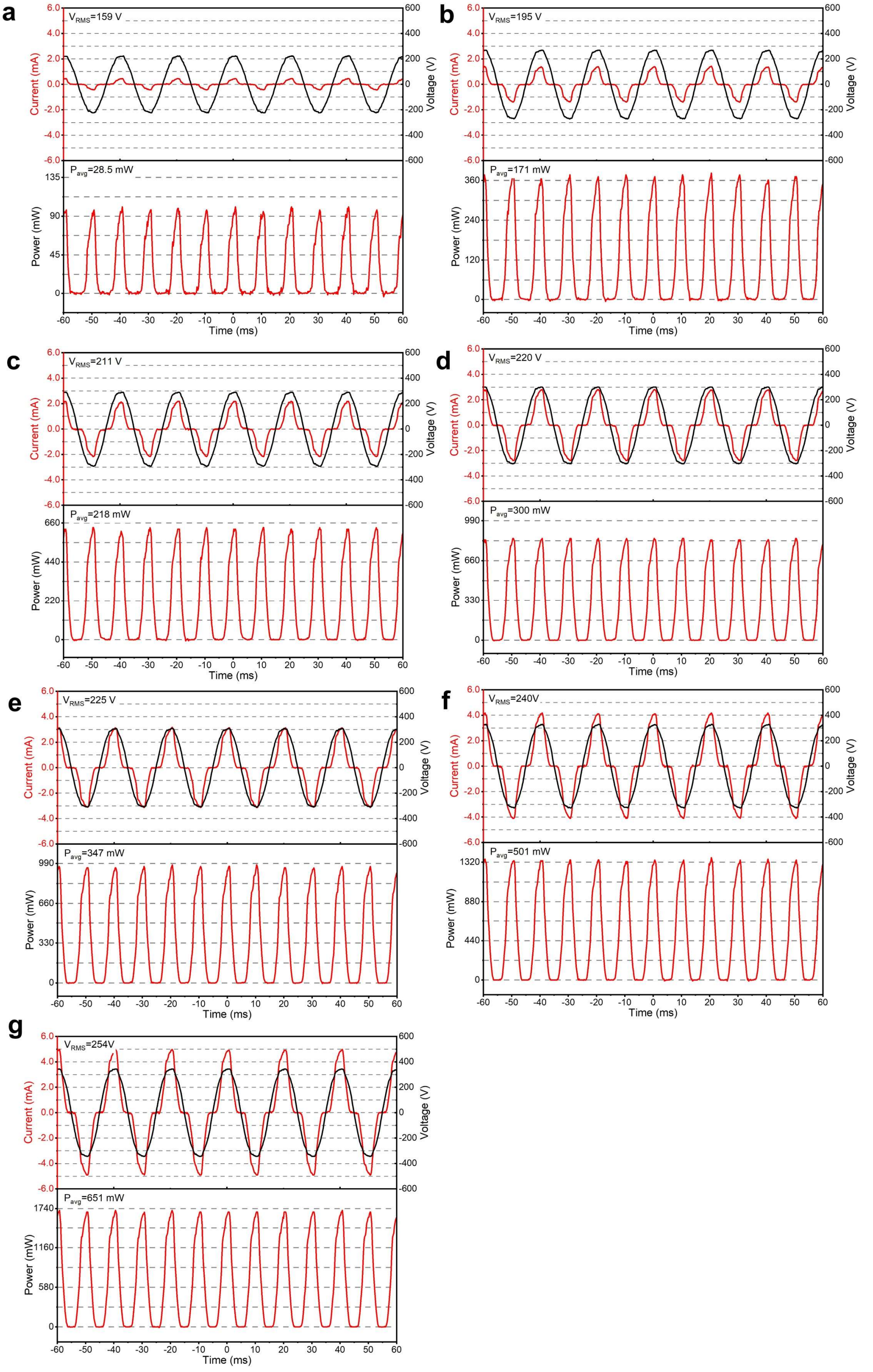

Supplementary Fig. 23 (PnP-QLED)$_{28}$ performance. a-g, *J*-*T*-*V* characterization and *P*-*T* characterization at V_{RMS}=159 V (P_{avg}=28.5 mW) (**a**), 195 V (P_{avg}=171 mW) (**b**), 211 V (P_{avg}=218 mW) (**c**), 220 V (P_{avg}=300 mW) (**d**), 225 V (P_{avg}=347 mW) (**e**), 240 V (P_{avg}=501 mW) (**f**), 254 V (P_{avg}=651 mW) (**g**), respectively.

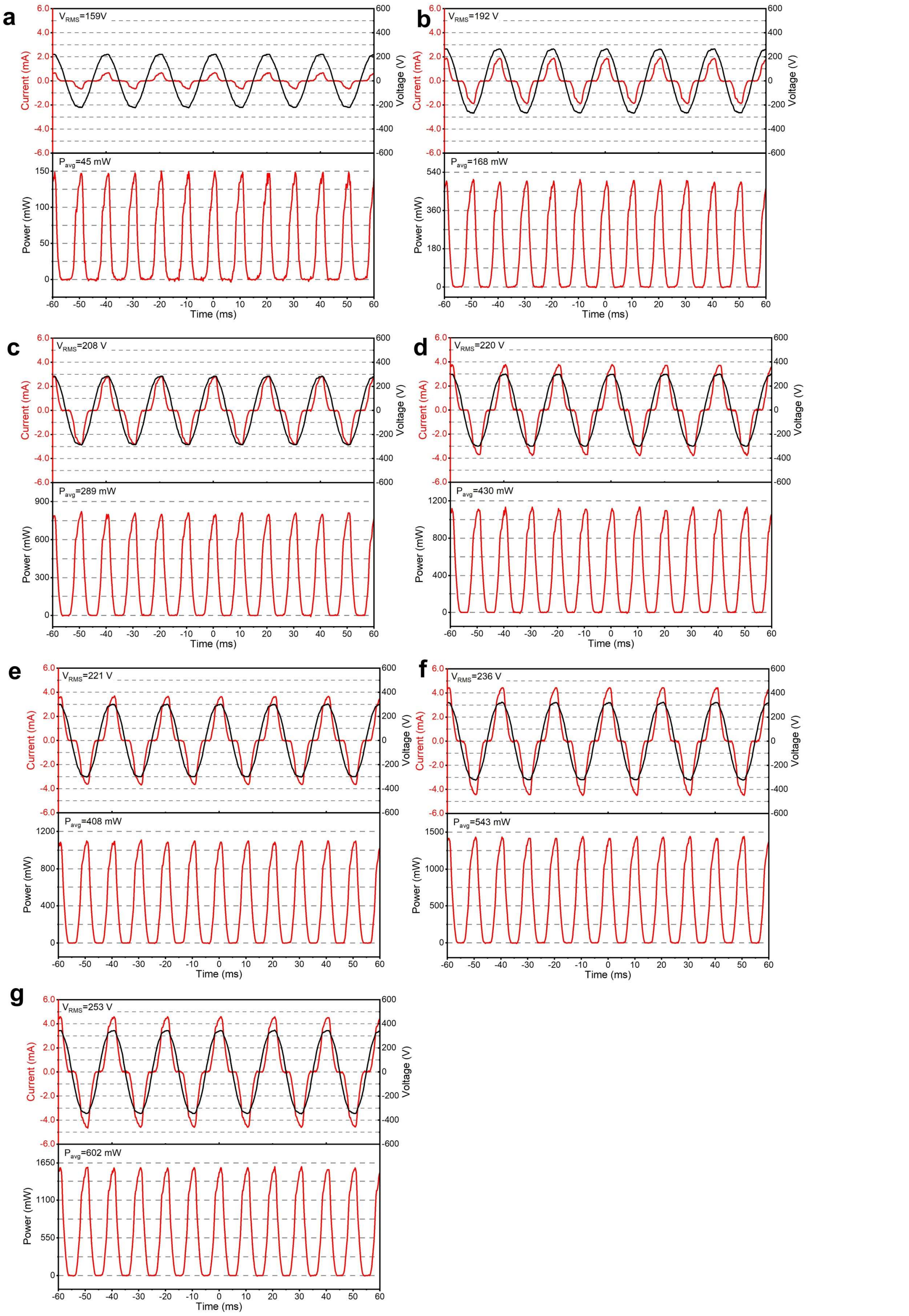

Supplementary Fig. 24 (PnP-QLED)$_{26}$ performance. a-g, *J*-*T*-*V* characterization and *P*-*T* characterization at V_{RMS}= 159 V (P_{avg}=45 mW) (**a**), 192 V (P_{avg}=168 mW) (**b**), 208 V (P_{avg}=289 mW) (**c**), 220 V (P_{avg}=430 mW) (**d**), 221 V (P_{avg}=408 mW) (**e**), 236 V (P_{avg}=543 mW) (**f**), 253 V (P_{avg}=602 mW) (**g**), respectively.

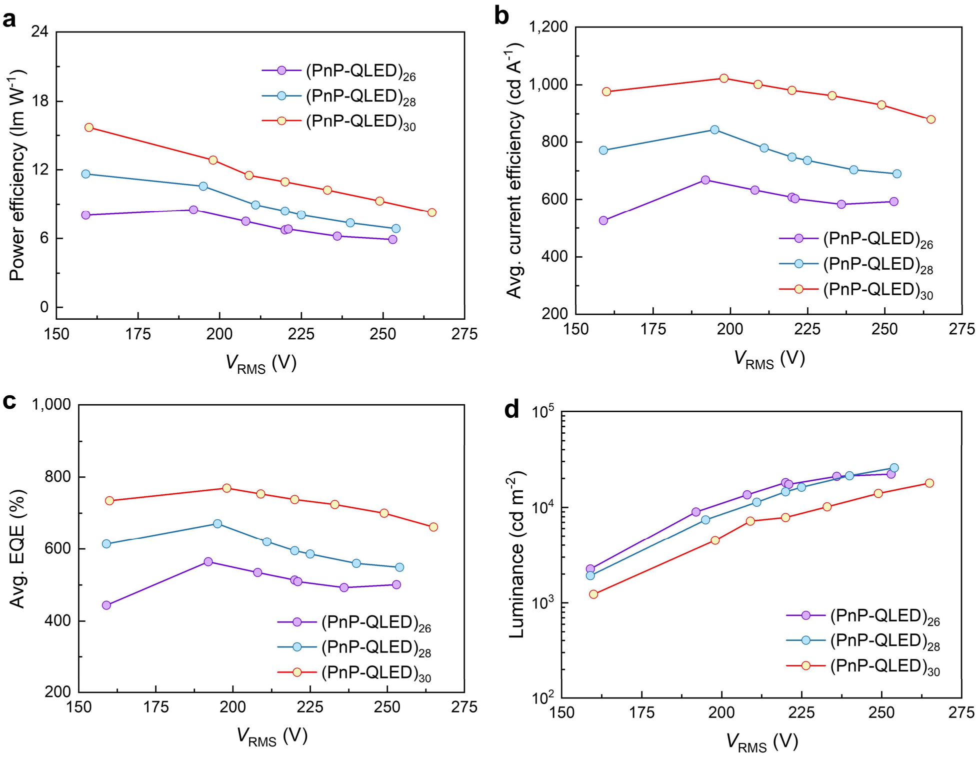

Supplementary Fig. 25 Performance of (PnP-QLED)*n*. The PE-V_{RMS} **a**, CE-V_{RMS} **b**, EQE-V_{RMS} **c**, and L-V_{RMS} characteristic **d**, curves of (PnP-QLED)$_n$ with n=26 (purple), 28 (blue), 30 (red), respectively. V_{RMS} is the root mean square voltage.

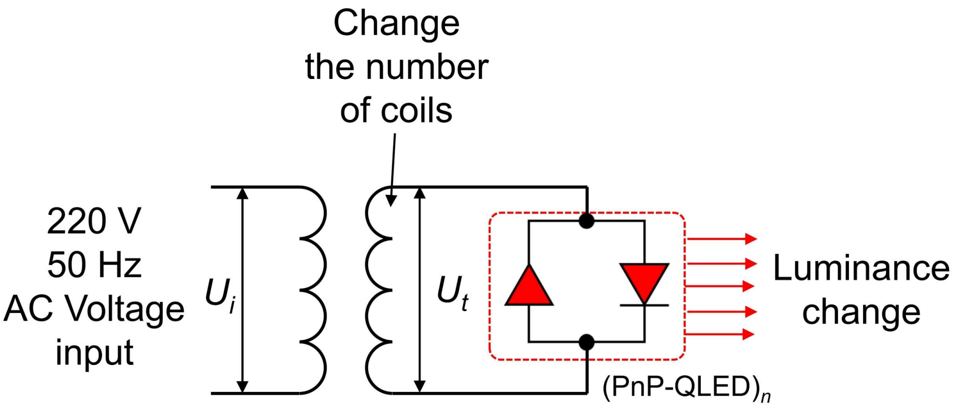

Supplementary Fig. 26 The luminance control setup for (PnP-QLED)$_n$. The AC voltage can be simply tuned by changing the number of coils in the AC transformer, thereby providing an easy and cost effective way for tuning the brightness of (PnP-QLED)$_n$.

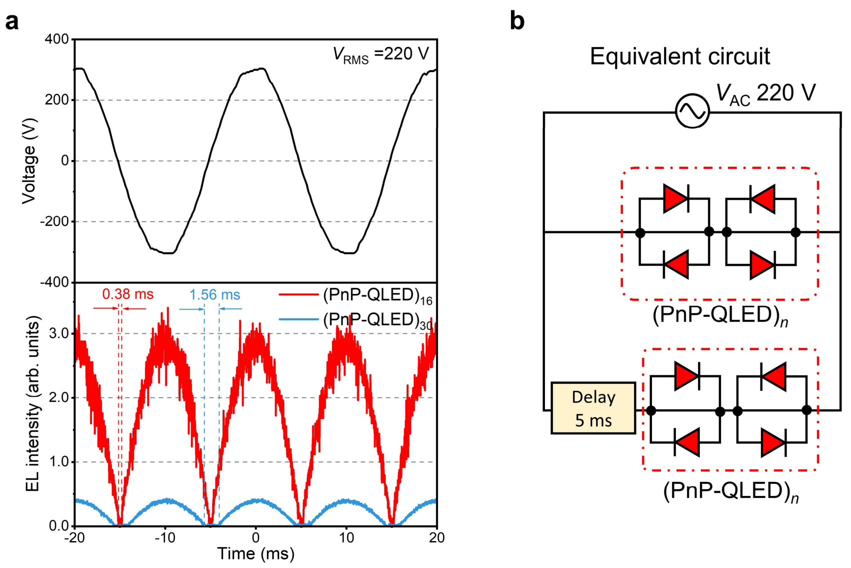

Supplementary Fig. 27 The TrEL of (PnP-QLED)*n* driven by 220 V/50 Hz household AC voltage. a, The 220 V/50 Hz household AC voltage and the time-resolved electroluminescence (TrEL) of $(PnP\text{-}QLED)_{16}$ (red) and $(PnP\text{-}QLED)_{30}$ (blue) directly driven by 220 V/50 Hz household AC power supply. **b**, Schematic circuit diagram of two (PnP-QLED)*n* under 220 V/50 Hz AC driving for reducing the luminance flickering.

Supplementary References:

1. Hwu, K. I., Tu, W. C. & Fang, Y. T. Dimmable AC LED driver with efficiency improved based on switched LED module. *J. Display Technol.* **10**, 171-181 (2014).
2. Zhang, F., Ni, J. & Yu, Y. High power factor AC-DC LED driver with film capacitors. *IEEE Trans. Power Electron.* **28**, 4831-4840 (2013).
3. Yu, W., Lai, J. S., Ma, H. & Zheng, C. High-efficiency DC-DC converter with twin bus for dimmable LED lighting. *IEEE Trans. Power Electron.* **26**, 2095-2100 (2011).
4. Malcovati, P., Belloni, M., Gozzini, F., Bazzani, C. & Baschirotto, A. A 0.18-μm CMOS, 91%-efficiency, 2-A scalable buck-boost DC-DC converter for LED drivers. *IEEE Trans. Power Electron.* **29**, 5392-5398 (2014).
5. Wood, V., Halpert, J. E., Panzer, M. J., Bawendi, M. G. & Bulović, V. Alternating current driven electroluminescence from ZnSe/ZnS:Mn/ZnS nanocrystals. *Nano Lett.* **9**, 2367-2371 (2009).
6. Perumal, A. et al. Novel approach for alternating current (AC)-driven organic light-emitting devices. *Adv. Funct. Mater.* **22**, 210-217 (2012).
7. Perumal, A., Lüssem, B. & Leo, K. High brightness alternating current electroluminescence with organic light emitting material. *Appl. Phys. Lett.* **100**, 103307 (2012).
8. Liu, S. Y., Chang, J. H., Wu, I. W. & Wu, C. I. Alternating current driven organic light emitting diodes using lithium fluoride insulating layers. *Sci. Rep.* **4**, 7559 (2014).
9. Zhang, J., Tsai, H., Nie, W. & Hu, B. Enabling AC electroluminescence in quasi-2D perovskites by uniformly arranging different-n-value nanoplates to allow bidirectional charge transport. *Nano Energy* **79**, 105413 (2021).
10. Fröbel, M. et al. Enhancing the efficiency of alternating current driven organic light-emitting devices by optimizing the operation frequency. *Org. Electron.* **14**, 809-813 (2013).

11. Wang, Z. G. et al. Flexible graphene-based electroluminescent devices. *ACS Nano* **5**, 7149-7154 (2011).
12. Sung, J. et al. AC field-induced polymer electroluminescence with single wall carbon nanotubes. *Nano Lett.* **11**, 966-972 (2011).
13. Lee, J. H. et al. A field-induced hole generation layer for high performance alternating current polymer electroluminescence and its application to extremely flexible devices. *J. Mater. Chem. C* **4**, 4434-4441 (2016).
14. Cho, S. H. et al. Extremely bright full color alternating current electroluminescence of solution-blended fluorescent polymers with self-assembled block copolymer micelles. *ACS Nano* **7**, 10809-10817 (2013).
15. Xia, F., Sun, X. W. & Chen, S. Alternating-current driven quantum-dot light-emitting diodes with high brightness. *Nanoscale* **11**, 5231-5239 (2019).
16. Chen, Y., Xia, Y., Smith, G. M. & Carroll, D. L. Frequency-dependent, alternating current-driven, field-induced polymer electroluminescent devices with high power efficiency. *Adv. Mater.* **26**, 8133-8140 (2014).
17. Chen, Y. et al. High-color-quality white emission in AC-driven field-induced polymer electroluminescent devices. *Org. Electron.* **15**, 182-188 (2014).
18. Chen, Y. et al. Solution-processed highly efficient alternating current-driven field-induced polymer electroluminescent devices employing high-k relaxor ferroelectric polymer dielectric. *Adv. Funct. Mater.* **24**, 1501-1508 (2014).
19. Xia, Y. et al. Alternating current-driven, white field-induced polymer electroluminescent devices with high power efficiency. *Org. Electron.* **15**, 3282-3291 (2014).
20. Zhang, X. et al. Color-tunable, spectra-stable flexible white top-emitting organic light-emitting devices based on alternating current driven and dual-microcavity technology. *ACS Photonics* **6**, 2350-2357 (2019).
21. Zhao, C. et al. Alternating-current-driven color-tunable organic light-emitting triodes. *Adv. Opt. Mater.* **9**, 2001655 (2021).
22. Kim, E. H. et al. Organic light emitting board for dynamic interactive display. *Nat.*

Commun. **8**, 14964 (2017).

23. Zhang, X., Liu, S., Zhang, L. & Xie, W. In-planar-electrodes organic light-emitting devices for smart lighting applications. *Adv. Opt. Mater.* **7**, 1703552 (2019).
24. Wang, T., Chen, Z., Zhang, H. & Ji, W. Color-tunable alternating-current quantum dot light-emitting devices. *ACS Appl. Mater. Interfaces* **13**, 45815-45821 (2021).
25. Ji, J. et al. Three-phase electric power driven electroluminescent devices. *Nat. Commun.* **12**, 54 (2021).
26. Fries, F., Fröbel, M., Lenk, S. & Reineke, S. Transparent and color-tunable organic light-emitting diodes with highly balanced emission to both sides. *Org. Electron.* **41**, 315-318 (2017).
27. Fröbel, M. et al. Get it white: color-tunable AC/DC OLEDs. *Light: Sci. Appl.* **4**, e247-e247 (2015).
28. Zhang, H., Su, Q. & Chen, S. Quantum-dot and organic hybrid tandem light-emitting diodes with multi-functionality of full-color-tunability and white-light-emission. *Nat. Commun.* **11**, 2826 (2020).
29. Zhang, H., Chen, L. & Chen, S. Quantum-dot and organic hybrid tandem light-emitting diodes with color-selecting intermediate electrodes for full-color displays. *Nanoscale* **13**, 16781-16789 (2021).
30. Neyts, K. A. Simulation of light emission from thin-film microcavities. *J. Opt. Soc. Am. A* **15**, 962-971 (1998).
31. Furno, M., Meerheim, R., Hofmann, S., Lüssem, B. & Leo, K. Efficiency and rate of spontaneous emission in organic electroluminescent devices. *Phys. Rev. B* **85**, 115205 (2012).
32. W. L. Barnes. Electromagnetic crystals for surface plasmon polaritons and the extraction of light from emissive devices. *J. Lightwave Technol.* **17**, 2170 (1999).
33. W. L. Barnes. Fluorescence near interfaces: The role of photonic mode density. *J. Mod. Opt.* **45**, 661-699 (1998).
34. Yuan, C., Tian, F. & Chen, S. ZnSeTe blue top-emitting QLEDs with color

saturation near Rec.2020 standards and efficiency over 18.16%. *Nano Res.* **16,** 5517-5524 (2023).
