# Supplementary material for: Household alternating current electricity plug-and-play quantum-dot light-emitting diodes
Source: Nat Commun. 2024 Apr 25;15:3512. doi: 10.1038/s41467-024-47891-4 (PMC11045821; doi:10.1038/s41467-024-47891-4)
Supplement: Supplementary file 3 — Description of Additional Supplementary Files [file 41467_2024_47891_MOESM3_ESM.pdf]

Description of Additional Supplementary Files

File Name: Supplementary Movie 1
Description: A tandem QLED with B-QLED and T-QLED connected in in parallel. Driven by an AC source, both B-QLED and T-QLED are alternately turned on.

File Name: Supplementary Movie 2
Description: A basis PnP-QLED with two tandem QLEDs connected in series. Driven by an AC source, the B-QLED (or T-QLED) of the first tandem device and the T-QLED (or B-QLED) of the second tandem device are simultaneously turned on.

File Name: Supplementary Movie 3
Description: A $(PnP\text{-}QLED)_{30}$ with 30 tandem QLEDs connected in series. By increasing the AC voltage level, the brightness of the device is gradually increased. Note that the observable flicker is due to the mismatch of the QLED lighting frequency and the frame captured frequency of the camera. The emission is quite stable observed by eyes.

File Name: Supplementary Movie 4
Description: The developed red, yellow, and white $(PnP\text{-}QLED)_{30}$ can be directly plugged into a household 220 V/50 Hz power supply without needing any accessories. Note that the observable flicker is due to the mismatch of the QLED lighting frequency and the frame captured frequency of the camera. The emission is quite stable observed by eyes.
